# The quality of reporting methods and results of cost-effectiveness analyses in Spain: a methodological systematic review

**DOI:** 10.1186/s13643-015-0181-5

**Published:** 2016-01-07

**Authors:** Ferrán Catalá-López, Manuel Ridao, Adolfo Alonso-Arroyo, Anna García-Altés, Chris Cameron, Diana González-Bermejo, Rafael Aleixandre-Benavent, Enrique Bernal-Delgado, Salvador Peiró, Rafael Tabarés-Seisdedos, Brian Hutton

**Affiliations:** Fundación Instituto de Investigación en Servicios de Salud, Valencia, Spain; Department of Medicine, University of Valencia/INCLIVA Health Research Institute and CIBERSAM, Valencia, Spain; Clinical Epidemiology Program, Ottawa Hospital Research Institute (OHRI), Ottawa, ON Canada; Instituto Aragonés de Ciencias de la Salud (I + CS), Red de Investigación en Servicios de Salud en Enfermedades Crónicas (REDISSEC), Zaragoza, Spain; Department of History of Science and Documentation, University of Valencia, Valencia, Spain; CIBERESP, Institut d’Investigació Biomèdica (IIB) Sant Pau, Barcelona, Spain; Evidence Synthesis Group, Cornerstone Research Group, Burlington, ON Canada; Division of Pharmacoepidemiology and Pharmacovigilance, Spanish Medicines and Healthcare Products Agency (AEMPS), Madrid, Spain; Ingenio-Spanish National Research Council (CSIC) and UISYS-University of Valencia, Valencia, Spain; FISABIO-Salud Pública, Red de Investigación en Servicios de Salud en Enfermedades Crónicas (REDISSEC), Valencia, Spain; School of Epidemiology, Public Health and Preventive Medicine, Faculty of Medicine, University of Ottawa, Ottawa, ON Canada

**Keywords:** Cost-effectiveness analysis, Cost-utility analysis, Quality-adjusted life years, Methodology, Reporting, Spain

## Abstract

**Background:**

Cost-effectiveness analysis has been recognized as an important tool to determine the efficiency of healthcare interventions and services. There is a need for evaluating the reporting of methods and results of cost-effectiveness analyses and establishing their validity. We describe and examine reporting characteristics of methods and results of cost-effectiveness analyses conducted in Spain during more than two decades.

**Methods:**

A methodological systematic review was conducted with the information obtained through an updated literature review in PubMed and complementary databases (e.g. Scopus, ISI Web of Science, National Health Service Economic Evaluation Database (NHS EED) and Health Technology Assessment (HTA) databases from Centre for Reviews and Dissemination (CRD), Índice Médico Español (IME) Índice Bibliográfico Español en Ciencias de la Salud (IBECS)). We identified cost-effectiveness analyses conducted in Spain that used quality-adjusted life years (QALYs) as outcome measures (period 1989–December 2014). Two reviewers independently extracted the data from each paper. The data were analysed descriptively.

**Results:**

In total, 223 studies were included. Very few studies (10; 4.5 %) reported working from a protocol. Most studies (200; 89.7 %) were simulation models and included a median of 1000 patients. Only 105 (47.1 %) studies presented an adequate description of the characteristics of the target population. Most study interventions were categorized as therapeutic (189; 84.8 %) and nearly half (111; 49.8 %) considered an active alternative as the comparator. Effectiveness of data was derived from a single study in 87 (39.0 %) reports, and only few (40; 17.9 %) used evidence synthesis-based estimates. Few studies (42; 18.8 %) reported a full description of methods for QALY calculation. The majority of the studies (147; 65.9 %) reported that the study intervention produced “more costs and more QALYs” than the comparator. Most studies (200; 89.7 %) reported favourable conclusions. Main funding source was the private for-profit sector (135; 60.5 %). Conflicts of interest were not disclosed in 88 (39.5 %) studies.

**Conclusions:**

This methodological review reflects that reporting of several important aspects of methods and results are frequently missing in published cost-effectiveness analyses. Without full and transparent reporting of how studies were designed and conducted, it is difficult to assess the validity of study findings and conclusions.

**Electronic supplementary material:**

The online version of this article (doi:10.1186/s13643-015-0181-5) contains supplementary material, which is available to authorized users.

## Background

Cost-effectiveness analysis has been recognized as an important tool to assist clinicians, scientists and policymakers in determining the efficiency of healthcare interventions, guiding societal decision-making on the financing of healthcare services and establishing research priorities. Given that the information provided by cost-effectiveness analysis has the potential to impact population health and health services, there is a need for evaluating the reporting of methods and results of cost-effectiveness analyses and establishing their validity to inform policymaking [1–4].

Diverse approaches to synthesize evidence have been considered in biomedical research [5–8], including economic evaluations of healthcare interventions [9–16]. At the same time, decision-making in health care requires an understanding of the state of economic evaluation at a national level, where the completeness of the reporting is generally less well understood but where specific priorities are often set. As a way of understanding the maturity and growth of the field, several smaller studies have examined a limited set of reporting characteristics of cost-effectiveness analyses published in Spain [17–20]. Spain was a pioneer in proposing the standardization and reporting of methodology applicable to cost-effectiveness analysis [21, 22]. However, the institutional and regulatory framework has so far not helped the application of the methodology to the public health decisions. The central government of Spain is the main decision-maker in pricing and reimbursement related to new medicines and healthcare technologies, although with a high decentralization of health jurisdictions in several regional health services, but traditionally, there have been no national requirements related to the cost-effectiveness for making coverage decisions.

We present herein a case study about reporting practices of economic evaluations of healthcare interventions in one Western European country: Spain. Specifically, this study expands upon previous research [23, 24] to comprehensively describe and examine reporting characteristics of methods and results of cost-effectiveness analyses conducted in Spain during more than two decades.

## Methods

This methodological systematic review has been reported according to the Preferred Reporting Items for Systematic Reviews and Meta-Analyses (PRISMA) statement [25] (see Additional file 1: Table S3). A brief protocol was developed prior to the initiation of this review. It can be acquired by request from the corresponding authors. We did not register the protocol with PROSPERO given that the register does not accept methodological reviews.

### Literature search

The results from a previous review that examined collaborative patterns of scientific production in a cohort of cost-effectiveness analyses conducted in Spain within the period 1989–2011 [23] were updated with the studies published until December 2014 and subsequently analysed. A systematic search was performed in PubMed/MEDLINE and other databases such as Scopus, ISI Web of Science, National Health Service Economic Evaluation Database (NHS EED) and Health Technology Assessment (HTA) databases of the Centre for Reviews and Dissemination (CRD) at the University of York, UK, as well as *Índice Médico Español* (IME) and *Índice Bibliográfico Español en Ciencias de la Salud* (IBECS). The search included a broad range of terms related to economic evaluations of healthcare interventions, cost-effectiveness analyses and the geographical area “Spain”. For the section of geographical area, the search was based on a previously validated filter by Valderas [26] to minimize bias regarding the indexing of geographical items. This filter is constructed around three complementary approaches: (a) the term “Spain” and its variants in various languages; (b) related mainly to region and province place names and (c) acronyms for regional health services. PubMed/MEDLINE and the above mentioned complementary databases were searched from January 1, 2011 to December 31, 2014; the PubMed/MEDLINE search strategy is provided in an online supplement to this review (see Additional file 1: Table S1). Furthermore, manual searches were made for publicly available reports from the Health Technology Agencies and publications in specialized Spanish journals.

### Inclusion criteria and study selection

Our selection of studies was based on cost-effectiveness analyses of healthcare interventions that used quality-adjusted life years (QALYs) as outcome measure (see Table 1 for terminology). In the health economic literature, this type of studies is sometimes known as “cost-utility analyses”. We selected this type of cost-effectiveness analyses because many scientists and policymakers have recommended the QALY framework as the standard reference for cost-effectiveness [27]. Studies had to be undertaken in the Spanish population. Review studies, editorials, and abstracts of congresses were excluded. If an article was found repeated in several publications, that published earlier (e.g. when there are two or more articles of the same study) and/or published in a journal with higher impact factor (e.g. when there exists a study published in both health technology assessment report and journal manuscript) was included.

**Table 1:** Terminology

**Cost-effectiveness analysis** is a specific form of economic evaluation comparing two or more alternative programmes by measuring costs and consequences. Consequences are measured in natural units (e.g. life years gained or cases averted). **Cost-utility analysis** is a variant of cost-effectiveness analysis, where consequences are measured in terms of summary measures of population health such as quality-adjusted life years. **Cost-effectiveness acceptability curve** is a graphical representation of the cost-effectiveness comparison between two interventions and plots the probability that one intervention is more cost-effective than other, as a function of the willingness-to-pay threshold for one additional unit of benefits. **Incremental cost-effectiveness ratio** (ICER) is the ratio of the change in costs of an intervention (compared to the alternative) to the change in effects of the intervention. **Quality-adjusted life years** (QALYs) are a measure that combines length of life and quality of life in a single outcome.	

All citations of potential relevance identified from the literature search were screened by one reviewer. Two reviewers reviewed all potentially relevant articles in full text. Final inclusion was confirmed if both reviewers felt the study was directly relevant to the objectives of this methodological review. Planned involvement of a third party to deal with unresolved discrepancies was not required.

### Data collection

Two reviewers (with expertise in health economics and evidence synthesis) extracted data from each retrieved paper independently. Data were collected using a self-developed item data collection form designed to assess reporting details of the studies. The process of data extraction was piloted in 20 records. A final data extraction form was then agreed. To enable description of the characteristics and the quality of reporting of cost-effectiveness analyses in each report, we gathered the following information from all studies: year and journal of publication, impact factor (according to 2014 Journal Citation Report), country of first author, mention of a protocol, study objective, study design (e.g. randomized trial, observational study, simulation model), intervention targeted (e.g. prevention, diagnosis/prognosis, treatment, rehabilitation), type of comparators (e.g. active alternative, do nothing or placebo, usual care), perspective of analysis (in terms of which costs are considered, e.g. society, national healthcare system, hospital, others), type of costs (e.g. direct or indirect) and sources of information, the main cause of disease to which the intervention or health programme was addressed, description of population characteristics, time horizon, sources of clinical effectiveness (e.g. based on a single study or based on systematic reviews and meta-analyses), full description of methods for QALY calculation, discussion of assumptions and validation of models (if applicable), discount rates for costs and outcomes, results for the primary outcome in the base case scenario (e.g. “more costs, more QALYs”, “less costs, more QALYs”, “less costs, comparable QALYs”), incremental analyses including incremental cost-effectiveness ratios (ICERs), uncertainty measures (e.g. confidence intervals, acceptability curves), sensitivity analyses, limitations of study, comparison of results with those of other studies, hypothetical willingness-to-pay threshold and study conclusions. Conclusions reported in the published article were defined as follows: favourable if the intervention was clearly claimed to be the preferred choice (e.g. cited as “cost-effective”, “reduced costs”, “produced cost savings”, “an affordable option”, “value for money”); unfavourable if the final comments were negative (e.g. the intervention is “unlikely to be cost-effective”, “produced higher costs”, “is economically unattractive” or “exceeded conventional thresholds of willingness to pay”); and neutral or uncertain when the intervention of interest did not surpass the comparator and/or when some uncertainty was expressed in the conclusions. Disclosures of funding source, conflicts of interest and authors’ contributions were also evaluated.

### Statistical analysis

A descriptive analysis was performed using frequency and percentage counts. All calculations were performed using Stata (Version 13, StataCorp LP, College Station, TX, USA).

## Results

### Search

The flow diagram in Additional file 1: Figure S1 presents the process of study selection.

Eight out of 131 identified studies from the cohort of cost-effectiveness analysis conducted within the period 1989–2011 [23] were excluded for not meeting the defined criteria. Our updated search identified 2014 records. Initial screening excluded 1914 records. The remaining 100 full-text articles were assessed for additional scrutiny, of which 21 where ineligible. Complementary searches through other sources (e.g. publicly available reports from the Health Technology Agencies and publications in specialized Spanish journals) identified 21 additional studies and were added to the previously identified, obtaining a total sample of 223 studies (see Additional file 1: Table S2).

### General characteristics

The 223 studies were published in 98 journals (206; 92.4 %) or assessment reports by the health technology assessment agencies (17; 7.6 %). The majority of the journals published only one cost-effectiveness analysis although 15 journals each published four or more studies (Table 2). Most studies were published in journals with impact factors ≤5.0 and only four studies were published in journals with impact factor >10. The number of studies increased exponentially over the study period (Additional file 1: Figure S2), with nearly half of the cost-effectiveness analyses published during 2011–2014 (110; 49.3 %). More than half (127; 57.0 %) of the reports were written in English. The studies included a median of six authors although 44 (19.7 %) were authored by eight or more authors and only 3 (1.3 %) reports were single authored. The majority of the interventions were classified as treatments (189; 84.8 %)—of which more than 75 % (143/189) were pharmaceuticals. Cardiovascular diseases (47; 21.1 %) and malignant neoplasms (36; 16.1 %) were the disease conditions most commonly studied.

**Table 2:** Characteristics of included cost-effectiveness analyses (*n* = 223)

Characteristic	Number	Percent
Journals publishing		
1 paper	60	61.2
2 papers	17	17.3
3 papers	6	6.1
4 papers or more	15	15.3
Papers by source		
Journal articles	206	92.4
Health technology assessment reports	17	7.6
Papers by journal impact factor (JCR 2014)		
None	88	39.5
0.1–2.0	61	27.3
2.1–5.0	58	26.0
5.1–10.0	12	5.4
>10.0	4	1.8
Papers by language of publication		
English	127	57.0
Spanish	96	43.0
Number of authors per paper		
1	3	1.3
2–3	33	14.8
4–7	143	64.8
≥8	44	19.7
Country of first author		
Spain	183	82.1
UK	8	3.6
USA	7	3.1
Italy	5	2.2
The Netherlands	4	1.8
Sweden	4	1.8
Other	12	5.4
Focus of interventions		
Prevention	18	8.1
Diagnosis/prognosis	15	6.7
Treatment	189	84.8
Rehabilitation	1	0.4
Disease conditions		
Cardiovascular diseases	47	21.1
Malignant neoplasms	36	16.1
Infectious diseases	31	13.9
Neurological and mental disorders	30	13.4
Musculoskeletal disorders	20	9.0
Other conditions	59	26.5

*JCR* Journal Citation Report 2014

### Reporting characteristics of methods and results

Table 3 provides a summary of the descriptive and reporting characteristics of the included studies. The majority of the study reports used the specific terms “cost-effectiveness” or “cost-utility analysis” in the title (181; 81.2 %) and presented clearly the study question (187; 83.9 %). However, only 10 studies (4.5 %) reported working from a protocol—of which 7 were randomized controlled trials, 2 were simulation models and 1 was an observational study.

**Table 3:** Descriptive and reporting characteristics of included cost-effectiveness analyses (*n* = 223)

Category	Characteristic	Number	Percent
Title	Identification		
	Specific terms “cost-effectiveness” or “cost-utility analysis” in title	181	81.2
Objective	Study question		
	Clear presentation of study question and its relevance for decision-making	187	83.9
Methods	Protocol		
	Existence of study protocol (or a priori established methods)	10	4.5
	Type of study		
	Model based	200	89.7
	Deterministic decision-tree model	29	13.0
	Markov model	135	60.5
	Discrete event simulation	11	4.9
	Other (or unclear)	25	11.2
	Non-model based	23	10.3
	Observational (non-interventional) study	13	5.8
	Randomized controlled trial	10	4.5
	Population		
	Number of participants included (or simulated)	127	57.0
	Adequate description of characteristics of the base case population	105	47.1
	Adults	170	76.2
	Children and adolescents	11	4.9
	Newborn and infants (less than 1 year)	8	3.5
	Overall population	4	1.8
	Not reported	30	13.5
	Type of interventions		
	Pharmaceuticals	143	64.1
	Device/procedure	28	12.6
	Screening	16	7.2
	Surgery	12	5.4
	Educational/behavioural	8	3.6
	Other	16	7.2
	Type of comparators		
	Active alternative	111	49.8
	Usual care	73	32.7
	Placebo or do nothing	39	17.5
	Adequate description of interventions and comparators	184	82.5
	Study perspective clearly stated	207	92.8
	National Health System only	156	70.0
	National Health System and societal	25	11.2
	Societal only	17	7.6
	Hospital	9	4.0
	Time horizon reported	218	97.8
	Short term	44	19.7
	Long term (>1 year and lifetime)	174	78.0
	Diagram of model or patients/events pathway reported	178	79.8
	Assumptions discussed	172	77.1
	Model validation discussed (when applicable)	88	44.0
	Reasons for the specific model used (when applicable)	91	45.5
	Measurement of effectiveness^a^		
	Based on a single study	87	39.0
	Based on evidence synthesis (e.g. systematic review and/or meta-analysis)	40	17.9
	Full description of QALY calculation	42	18.8
	Harms were considered	129	57.8
	Cost and resources information		
	Source of valuation for all cost items reported	216	96.9
	Quantity of resources	107	48.0
	Year of monetary units	195	87.4
	Costing		
	Direct costs only	182	81.6
	Direct and indirect costs	41	18.4
	Discount rate for costs and QALYs	161	72.2
Results	Net costs reported	197	88.3
	Net benefits reported	192	86.1
	Incremental cost-effectiveness ratio (ICER) reported	207	92.8
	Confidence intervals (e.g. 95 % CI)	27	12.1
	Cost-effectiveness plane	99	44.4
	Acceptability curves	92	41.3
	Sensitivity analysis reported	201	90.1
	For costs	170	76.2
	For estimates of effectiveness	158	70.9
	For utility weights	95	42.6
	For discount rates	82	36.8
	Type of sensitivity analysis		
	Deterministic univariate	85	38.1
	Deterministic multivariate	6	2.7
	Probabilistic	110	49.3
	Results for the primary outcome in the base case scenario		
	More costs, more QALYs	147	65.9
	Less costs, more QALYs	63	28.3
	Less costs, comparable QALYs	5	2.2
	More costs, comparable QALYs	4	1.8
	Less costs, less QALYs	2	0.9
	Comparable costs, more QALYs	2	0.9
Discussion	Limitations of study discussed	197	88.3
	Results compared with those of other economic evaluations	165	74.0
	Hypothetical willingness-to-pay (WTP) threshold reported		
	<30,000 €/QALY	4	1.8
	30,000 €/QALY	126	56.5
	>30,000 €/QALY–≤50,000 €/QALY	36	16.1
	>50,000 €/QALY	7	3.1
	Unclear or not reported	50	22.4
	Study conclusions		
	Favourable	200	89.7
	Unfavourable	12	5.4
	Neutral/unclear	11	4.9
Other	Disclosed funding sources	169	75.8
	Private/for profit	135	60.5
	Public	38	17.0
	None/not reported	49	22.0
	Mixed	1	0.4
	Disclosed conflicts of interest	135	60.5
	With conflicts of interest	94	42.1
	With no conflicts of interest	41	18.4
	Disclosed authors’ contribution	46	20.6

^a^Measurement of effectiveness only relates to effect estimates. The epidemiology of disease and/or transition probabilities were not necessarily based on a systematic review and meta-analysis

Of the identified studies, 200 (89.7 %) were model-based being Markov models as the most frequently reported (135; 60.5 %). A minimal number of non-model-based studies were randomized controlled trials (10; 4.5 %).

Overall, most of the analyses were conducted in the adult population (170; 76.2 %) but only 105 (47.1 %) presented an adequate description of the characteristics of the base case population or identified the indication clearly. The studies reporting the sample size (127; 57.0 %) included a median of 1000 patients (25th percentile = 301; 75th percentile = 10000), although this number varied considerably by the type of the study (e.g. clinical trials, median = 115 patients; observational studies, median = 200 patients; and simulation models, median = 1000 patients). Most of the studies included an adequate description of the interventions and comparators (184; 82.5 %). Nearly half (111; 49.8 %) of the studies considered an active alternative as the comparator (e.g. drug, device, procedure, programme), 73 (32.7 %) used usual care and 39 (17.5 %) placebos or “do nothing”. The study perspective was clearly stated in most of the analyses (207; 92.8 %). The national healthcare system perspective was the most commonly used (156; 70.0 %).

The time horizon was clearly reported in the majority of studies (218; 97.8 %). Overall, 174 studies (78.0 %) used a time horizon greater than 1 year.

Most studies (178; 79.8 %) reported on the diagram of modelling or flow of patients (e.g. in the case of randomized controlled trials and observational studies). Most studies (172; 77.1 %) reported on the assumptions adopted for the analyses. Regarding the simulation and modelling-based studies, nearly half reported reasons for the specific model used (91/200; 45.5 %) and/or provided some information on the model validation (88/200; 44.0 %) such as previous publication in other settings.

Effectiveness of data was derived from a single study in 87 (39.0 %) analyses. Only 40 (17.9 %) used evidence synthesis-based estimates (e.g. systematic reviews and meta-analyses).

The methods that were reported for calculating QALYs are detailed in Table 4. Overall, a small number of the studies (42; 18.8 %) reported a full description of methods for QALY calculation. About half of the studies (109; 48.9 %) reported information on the health-state classification system, of which the EuroQoL-5D was the instrument most commonly reported (82; 36.8 %). Half of the studies (115; 51.6 %) provided the source of the preferences. Most frequently, the patients and their caregivers (103; 46.2 %) were the source. Only a small number of the studies (43; 19.3 %) provided information on the measurement technique used for valuing health states. The time tradeoff (22; 9.9 %) was the most commonly used technique. The majority of the studies used the published international literature for data on utility weights (143; 64.1 %) and only 50 studies (22.4 %) reported country-specific utility weights for Spain.

**Table 4:** Descriptive and reporting characteristics of methods used in calculating QALYs

Category	Characteristic	Number	Percent
Health-state classification system	EuroQoL-5D	82	36.8
	SF-36	6	2.7
	Rosser scale	6	2.7
	Health Utility Index (HUI)	2	0.9
	Other	13	5.8
	Not reported	114	51.1
Source of preferences	Patient/caregiver	103	46.2
	Community	10	4.5
	Clinician/author	2	0.9
	Not reported	108	48.4
Measurement technique used for valuing health state	Time tradeoff (TTO)	22	9.9
	Visual analogue scale (VAS)	12	5.4
	Standard gamble (SG)	5	2.2
	Tariffs for classification	4	1.8
	Not reported	182	81.6
Country/region of reference for utility weights	National/local population (e.g. Spain)	50	22.4
	Citation of the international literature	143	64.1
	Not reported	30	13.5

Half of the studies (129; 57.8 %) reported on some aspect of harms.

Ninety-seven percent (216) of the studies identified sources of valuation for costing items, and 87.4 % (195) indicated the year of currency. Overall, 107 (48.0 %) studies described quantity of resources. Eighteen percent (41) of studies included indirect costs. Seventy-two percent (161) of studies discounted both costs and QALYs. Of the studies with a time horizon greater than 1 year (Table 5), the most commonly used was a 3 % discount rate.

**Table 5:** Discount rates used in included cost-effectiveness analyses

	Costs		QALYs	
Discount rate (%)	Number	Percent	Number	Percent
1.5	–	–	3	1.7
2	1	0.6	1	0.6
3	107	61.5	109	62.6
3.5	31	17.8	31	17.8
4	1	0.6	1	0.6
5	5	2.9	4	2.3
6	13	7.5	11	4.9
Total^a^	174	100.0	174	100.0

^a^Includes cost-effectiveness analyses that investigated costs or QALYs over time horizons of more than 1 year

In terms of results (Table 3), most of the studies (207; 92.8 %) reported ICERs (median = 16,908 €; 25th percentile = 8,998 €; 75th percentile = 38,000 €). However, few studies (27; 12.1 %) described point estimates together with an associated confidence interval. Nearly half of the studies (99; 44.4 %) reported the cost-effectiveness plane. Similarly, less than half of the studies (92; 41.3 %) reported a willingness-to-pay curve (“cost-effectiveness acceptability curve”) to contrast the results of the analyses against an arbitrary efficiency threshold. Overall, 90.1 % (201) of studies reported sensitivity analyses. About half of the studies (110; 49.3 %) conducted a probabilistic sensitivity analysis. The majority of the studies (147; 65.9 %) reported that the study intervention produced “more costs and more QALYs” than the alternative comparator for the primary outcome of the base case scenario. Sixty-three (28.3 %) studies reported that the intervention was a dominant strategy, that means that the study intervention was “more effective and less costly” than the alternative.

Overall, the vast majority of the studies (197; 88.3 %) discussed limitations of the analyses. Most studies (165; 74.0 %) compared their results with those of previous economic analyses. About half of the studies (126; 56.5 %) mentioned a hypothetical willingness-to-pay threshold of 30,000 €/QALY. The majority of studies (200; 89.7 %) reported favourable conclusions for the primary outcomes. Only a minority (12; 5.4 %) of published cost-effectiveness analyses reported unfavourable conclusions. About three fourths (169; 75.8 %) reported funding sources, being the private for-profit sector the main source (135; 60.5 %). Conflicts of interest were not disclosed in 88 (39.5 %) studies. Authors’ contributions were only reported in 46 (20.6 %) studies (Table 3).

## Discussion

In this methodological systematic review, we identified 223 reports of cost-effectiveness analyses conducted in Spain over the period 1989–2014. Overall, the studies covered a wide range of disease conditions but predominantly addressed questions about the efficiency of therapeutic interventions. Our review, as well as other previously published reviews [11–14], showed that the quality of reporting of cost-effectiveness analyses varies widely and, in many cases, essential components of reporting methods and results were missing in published reports, such as the use of study protocols, the adequate description of patient characteristics, the measurement of clinical effectiveness using a systematic review process or the adequate description of QALY calculation.

Our study suggests the need for improvement in several aspects of published cost-effectiveness analyses. An important element in assessing research conduct and reporting is the study protocol. As showed in this review, only 4 % of studies reported working from a protocol. Study protocols play an essential role in planning, conduct, interpretation, and external review of primary studies, but also in evidence synthesis of primary research. For example, the preparation and publication of a well-written protocol may reduce arbitrariness in decision-making when extracting and using data from primary research for populating health economic models. When clearly reported protocols are made available, they enable readers to identify deviations from planned methods and whether they bias the interpretation of results and conclusions [28, 29]. International registries (such as ClinicalTrials.gov for clinical trials and PROSPERO for systematic reviews and meta-analysis) are now a reality. Similarly, in recent years, reporting guidelines for protocols have been endorsed and implemented (e.g. Standard Protocol Items: Recommendations for Interventional Trials (SPIRIT) for protocols of clinical trials [28] and PRISMA for protocols (PRISMA-P) of systematic reviews [29]). However, in view of our results, this revolution has not occurred yet in the field of cost-effectiveness research and, thus, could warrant further pragmatic action.

Many cost-effectiveness analyses (about 53 %) did not report detailed information on baseline clinical characteristics (e.g. eligibility and exclusion criteria of participants, the severity of disease, the stage in the natural history of the disease, comorbidities). Inadequate reporting of the characteristics of the target population is a far greater barrier to the assessment of the study’s generalizability (applicability) and relevance to decision-making [30–32]. It is possible that this poor reporting reflects a major problem in secondary publications, such as many cost-effectiveness analyses using simulation models. Given that a clear understanding of these elements is required to judge to whom the results of a study apply (as the Consolidated Standards of Reporting Trials (CONSORT) [31, 32] statement underlines for randomized controlled trials), this information should also be provided in the report of cost-effectiveness analyses (e.g. in main text or in online supplement when allowed).

The vast majority (about 90 %) of published cost-effectiveness analyses used decision modelling as the main methodology. Decision modelling is considered a methodological approach of evidence synthesis that reaches beyond the scope of systematic reviews and meta-analyses. It is essential for cost-effectiveness research to use all relevant evidence on the effectiveness of interventions under evaluation. Rarely will all relevant evidence come from a single study, and typically, it will have to be drawn from several clinical studies [33]. A disappointing result of this review is that few published studies reported the use of a systematic review process for the measurement of clinical effectiveness. While systematic reviews and meta-analyses are considered to be the gold standard in knowledge synthesis, only 18 % of published cost-effectiveness analyses used evidence synthesis-based estimates of effect. Instead, 43 % of the studies make arbitrary decisions about what studies to use to inform effectiveness data, whereas 39 % of the studies reported that the effectiveness data derived from a single study (generally, without a clear description of why the single study was a sufficient source of all relevant clinical evidence). The use of QALYs is recognized as the main valuation technique to measure health outcomes in cost-effectiveness analyses. However, in our review, it was also troubling that few studies (19 %) reported a full description of methods for QALY estimation, thus potentially impairing confidence in the results and conclusions. Future studies should be transparent in reporting these important aspects.

Strong evidence of publication bias and other potential sources of bias have been reported in biomedical research [34–38]. For example, randomized controlled trials with “positive” findings are published more often, and more quickly, than trials with “negative” findings [37]. Similarly, empirical studies have detected that most published cost-effectiveness analyses report favourable findings [38]. In our review, very few published studies (about 10 %) reported unfavourable or neutral conclusions. Although it is somewhat premature to comment on this finding, this could be indicative of potential biases, such as publication bias or even potential screening a priori that may have been performed by the producers of studies, which would make that cost-effectiveness analyses would have been only conducted in cases where a “positive” result was expected. In our opinion, this issue requires further investigation.

Several reporting guidelines are available and endorsed for many types of biomedical research [25, 28, 29, 31, 39] but also for cost-effectiveness analyses [40–46]. Such tools promote the consistent reporting of a minimal set of information for scientists and researchers reporting studies and the editors and peer reviewers assessing them for publication. Endorsement of reporting guidelines by journals for randomized controlled trials [47] and systematic reviews [48] has been shown to improve the quality of reporting. The incorporation of reporting guidelines within the peer-review process could potentially contribute to improvements in the quality of reports of cost-effectiveness analyses. On this regard, the Consolidated Health Economic Evaluation Reporting Standards (CHEERS) statement [40] has been proposed as an attempt to consolidate and update previous efforts [41–46] into a single useful reporting guideline for cost-effectiveness research. Authors, peer reviewers and editors can promote reporting guideline endorsement and implementation as an important way to improve transparency and completeness of what they published, reducing waste in reporting research and increasing value [49, 50] of cost-effectiveness research.

Our study has several limitations. First, although the review has been drawn from an exhaustive review of original reports of cost-effectiveness analyses, it is possible that the search missed some articles with relevant elements or that some studies conducted may not have been published. In addition, for some reports repeated in several publications, our approach was the inclusion of those published in a journal with higher impact factor and/or published earlier [23, 24]. Thus, the decision to use report level instead treating the study as the unit of analysis may have limited the collection of all the reporting characteristics from multiple reports of the same study (where there exist). Second, we restricted our analysis to cost-effectiveness analyses that used QALYs as health outcome measure (namely, cost-utility analyses). In a previous descriptive analysis of economic evaluations conducted in Spain [24], only about 15 % are cost-utility analyses. It would be interesting to explore whether other forms of economic evaluations using alternative outcome measures results in similar reporting patterns. Third, we relied upon the expertise and experience of our authorship team and on existing documents [16, 22] to identify core items related to the conduct and reporting that we would like to see (in the position of potential readers) in any published cost-effectiveness analysis. Given the dynamic nature of research, some opportunities for future research and development could be the impact assessment of a specific reporting guideline (such as the CHEERS statement [40]) and/or local recommendations on the reporting quality of published studies [51]. Fourth, the extent of the reporting of cost-effectiveness analyses was limited to the information publicly available in the corresponding report (and online data supplements when available). There were no further inquiries or attempts to verify the data sources and tools used in the studies and only information about reporting characteristics was taken into account in the review, without considering other possible sources (e.g. contacting authors and/or their sponsors).

## Conclusions

We presented a national case study for more generalizable discussions about quality and transparency issues of reporting cost-effectiveness analyses, likely to be of interest to authors, peer reviewers and editors—but also research funders and regulators—both within and beyond Spain. Based on the existing evidence, several deficiencies in the reporting of important aspects of methods and results are apparent in published cost-effectiveness analyses.

Our study raises challenges for increasing value and reducing waste in cost-effectiveness research. Without full and transparent reporting of how studies were designed and conducted, it is difficult to assess validity of study findings and conclusions of published studies. This review also reinforces the need to improve mechanisms of peer review and publication process of cost-effectiveness research.

## Additional file

Additional file 1:
**Reporting methods and results of CEA Spain_annex.** **Table S1.** PubMed/MEDLINE search strategy. **Table S2.** Included studies. **Table S3.** PRISMA Checklist. **Figure S1.** Flow diagram for study selection. **Figure S2.** Growth in published cost-effectiveness analyses. Spain, 1989-2014.

## Abbreviations

CHEERS: Consolidated Health Economic Evaluation Reporting Standards
CONSORT: Consolidated Standards of Reporting Trials
CRD: Centre for Reviews and Dissemination
HTA: Health Technology Assessment
HUI: Health Utility Index
IBECS: Índice Bibliográfico Español en Ciencias de la Salud
ICER: incremental cost-effectiveness ratio
IME: Índice Médico Español
JCR: Journal Citation Report
NHS EED: NHS Economic Evaluation Database
PRISMA: Preferred Reporting Items for Systematic Reviews and Meta-Analyses
PRISMA-P: Preferred Reporting Items for Systematic Reviews and Meta-Analyses extension for Protocols
QALYs: quality-adjusted life years
SF-36: 36-Item Short Form Health Survey
SG: standard gamble
SPIRIT: Standard Protocol Items: Recommendations for Interventional Trials
TTO: time tradeoff
VAS: visual analogue scale
