# Supplementary material for: The quality of reporting methods and results of cost-effectiveness analyses in Spain: a methodological systematic review
Source: Syst Rev. 2016 Jan 7;5:6. doi: 10.1186/s13643-015-0181-5 (PMC4731991; doi:10.1186/s13643-015-0181-5)
Supplement: Additional file 1: — Reporting methods and results of CEA Spain_annex. Table S1. PubMed/MEDLINE search strategy. Table S2. Included studies. Table S3. PRISMA Checklist. Figure S1. Flow diagram for study selection. Figure S2. Growth in published cost-effectiveness analyses. Spain, 1989-2014. [file 13643_2015_181_MOESM1_ESM.doc]

**Annex.**

**Table S1. PubMed/MEDLINE search strategy.**

| **Cost-effectiveness terms:** Costs and cost analysis[mh] OR "cost-benefit analysis"[mh] OR "cost allocation"[mh] OR "cost control"[mh] OR "cost of illness"[mh] OR "cost savings"[mh] OR "cost sharing"[mh] OR "health expenditures"[mh] OR cost[ti] OR costs[ti] OR econom*[mh] OR econom*[ti] OR econom*[sh] OR saving*[ti] OR "fees and charges"[mh] OR reimburs*[ti] OR budget*[ti] OR expenditur*[ti] OR price[ti] or prices[ti] or pricing[ti] or pharmacoeconomic*[ti] OR finan*[ti] |
| --- |
| **Geographical terms:** ("Spain"[MeSH Terms] OR spain[Text Word]) OR espagne[All Fields] OR espana[All Fields] OR (spain[ad] OR espagne[ad] OR espana[ad]) OR osasunbidea[ad] OR osakidetza[ad] OR insalud[ad] OR sergas[ad] OR (catalunya[ad] OR catalonia[ad] OR catalogne[ad] OR cataluna[ad] OR catala[ad] OR (barcelon[ad] OR barcelona[ad] OR barcelone[ad] OR barcelones[ad] OR barceloneta[ad]) OR tarragona[ad] OR lleida[ad] OR lerida[ad] OR girona[ad] OR gerona[ad] OR sabadell[ad] OR hospitalet[ad] OR l'hospitalet[ad]) OR ((valencia[ad] OR valenciana[ad] OR valenciano[ad]) OR (castello[ad] OR castellon[ad]) OR alacant[ad] OR (alicant[ad] OR alicante[ad]) OR (murcia[ad] OR murcian[ad] OR murciana[ad] OR murciano[ad])) OR ((andalucia[ad] OR andaluciajunta[ad] OR andalusia[ad] OR andalusian[ad] OR andaluz[ad] OR andaluza[ad]) OR (sevill[ad] OR sevilla[ad] OR seville[ad]) OR (granada[ad] OR granade[ad]) OR huelva[ad] OR almeria[ad] OR cadiz[ad] OR jaen[ad] OR malaga[ad] OR (cordoba[ad] NOT (argentina[ad]) OR (extremadura[ad] OR caceres[ad] OR badajoz[ad] OR madrid[ad]) OR (castilla[ad] OR salamanca[ad] OR zamora[ad] OR valladolid[ad] OR segovia[ad] OR soria[ad] OR palencia[ad] OR avila[ad] OR burgos[ad]) OR (leon[ad] NOT (france[ad] OR clermont[ad] OR rennes[ad] OR lyon[ad] OR USA[ad] OR (mexic[ad] OR mexica[ad] OR mexican[ad] OR mexicana[ad] OR mexicano[ad] OR mexicanos[ad] OR mexico[ad]))) OR (galicia[ad] OR gallego[ad] OR compostela[ad] OR vigo[ad] OR coruna[ad] OR ferrol[ad] OR orense[ad] OR ourense[ad] OR pontevedra[ad]) OR (oviedo[ad] OR gijon[ad] OR (asturia[ad] OR asturiano[ad] OR asturias[ad] OR asturias'[ad])) OR ((cantabria[ad] OR cantabrico[ad] OR cantabro[ad]) OR santander[ad]) OR (vasco[ad] OR euskadi[ad] OR basque[ad] OR bilbao[ad] OR bilbo[ad] OR (donosti[ad] OR donostia[ad]) OR vizcaya[ad] OR guipuzcoa[ad] OR gipuzkoa[ad] OR alava[ad] OR araba[ad] OR vitoria[ad]) OR ((navarra[ad] OR navarro[ad]) OR pamplona[ad] OR iruna[ad] OR irunea[ad]) OR ((aragon[ad] OR aragones[ad]) OR zaragoza[ad] OR teruel[ad] OR huesca[ad]) OR (mancha[ad] OR ciudad real[ad] OR albacete[ad] OR cuenca[ad]) OR (toledo[ad] NOT (ohio[ad] OR us[ad] OR usa[ad] OR OH[ad])) OR (guadalajara[ad] NOT (mexic[ad] OR mexica[ad] OR mexican[ad] OR mexicana[ad] OR mexicano[ad] OR mexicanos[ad] OR OR mexico[ad])) OR ((balear[ad] OR balearen[ad] OR baleares[ad] OR balearic[ad] OR balears[ad] OR balearse[ad]) OR mallorca[ad] OR menorca[ad] OR ibiza[ad] OR eivissa[ad]) OR (palmas[ad] OR lanzarote[ad] OR (canaria[ad] OR canarian[ad] OR canarias[ad] OR canario[ad]) OR tenerife[ad]) |
| **Limits:** ("2011/01/01"[PDAT] : "2014/12/31"[PDAT]) |

**Table S2. Included studies.**

| 1. Villa G, Hernandez-Pastor LJ, Guix M, Lavernia J, Cuesta M. Cost effectiveness analysis of pazopanib in second-line treatment of advanced soft tissue sarcoma in Spain. Clin Transl Oncol 2014. doi: 10.1007/s12094-014 1191-9. Epub 2014 Jul 1. 2. Vilaprinyo E, Forne C, Carles M, Sala M, Pla R, Castells X, et al. Cost-effectiveness and harm-benefit analyses of risk-based screening strategies for breast cancer. PLoS One 2014;9(2):e86858. 3. Vallejo-Torres L, Castilla I, Gonzalez N, Hunter R, Serrano-Perez P, Perestelo-Perez L. Cost-effectiveness of electroconvulsive therapy compared to repetitive transcranial magnetic stimulation for treatment-resistant severe depression: a decision model. Psychol Med 2014:1-12. 4. Slof J, Diez Valle R, Galvan J. Cost-effectiveness of 5-aminolevulinic acid-induced fluorescence in malignant glioma surgery. Neurologia 2014 Jan 24. pii: S0213-4853(13)00286-7. doi: 10.1016/j.nrl.2013.11.002. [Epub ahead of print] 5. Segui MA, Crespo C, Cortes J, Lluch A, Brosa M, Becerra V, et al. Genomic profile of breast cancer: cost-effectiveness analysis from the Spanish National Healthcare System perspective. Expert Rev Pharmacoecon Outcomes Res 2014:1-11. 6. San Miguel R, Gimeno-Ballester V, Blazquez A, Mar J. Cost-effectiveness analysis of sofosbuvir-based regimens for chronic hepatitis C. Gut 2014 Oct 13. pii: gutjnl-2014-307772. doi: 10.1136/gutjnl-2014-307772. [Epub ahead of print] 7. Sagarra R, Costa B, Cabre JJ, Sola-Morales O, Barrio F. Lifestyle interventions for diabetes mellitus type 2 prevention. Rev Clin Esp (Barc) 2014;214(2):59-68. 8. Ribera A, Slof J, Andrea R, Falces C, Gutierrez E, Del Valle-Fernandez R, et al. Transfemoral transcatheter aortic valve replacement compared with surgical replacement in patients with severe aortic stenosis and comparable risk: Cost-utility and its determinants. Int J Cardiol 2014;182C:321-28. 9. Raspall-Chaure M, Martinez-Bermejo A, Sanchez-Carpintero R, Ruiz-Falco Rojas ML, Verdu-Perez A, Smeyers-Dura P, et al. [Cost-effectiveness of buccal midazolam in the treatment of prolonged convulsive seizures in the outpatient setting in Spain]. Rev Neurol 2014;58(11):481-6. 10. Nieves D, Puig-Peiro R, Ferrandiz C, Plazas MJ, Brosa M. Cost-effectiveness analysis of 5-fluorouracil 0.5%/salicylic acid 10% in the treatment of actinic keratosis in Spain. Expert Rev Pharmacoecon Outcomes Res 2014:1-5. 11. Molina-Cuadrado E, Mateo-Carras H, Nieto-Guindo P, Rodríguez-Gómez P. Coste-efectividad a largo plazo de ticagrelor frente a clopidogrel en síndrome coronario agudo en España. Farm Hosp 2014;38(4):10. 12. Luciano JV, D’Amico F, Cerda-Lafont M, Penarrubia-Maria MT, Knapp M, Cuesta-Vargas AI, et al. Cost-utility of cognitive behavioural therapy versus U.S. food and drug administration recommended drugs and usual care in the treatment of patients with fibromyalgia: an economic evaluation alongside a 6-month randomised controlled trial. Arthritis Res Ther 2014;16(5):451. 13. Levy AN, Garcia AR, Garcia-Agua NS, Hidalgo MV. Cost-effectiveness of omalizumab in severe persistent asthma in spain: a real-life perspective. J Asthma 2014:1-26. 14. Lee D, Wilson K, Akehurst R, Cowie MR, Zannad F, Krum H, et al. Cost-effectiveness of eplerenone in patients with systolic heart failure and mild symptoms. Heart 2014;100(21):1681-7. doi: 10.1136/heartjnl-2014-305673. Epub 2014 Jul 3. 15. Jodar-Sanchez F, Ortega F, Parra C, Gomez-Suarez C, Bonachela P, Leal S, et al. Cost-utility analysis of a telehealth programme for patients with severe chronic obstructive pulmonary disease treated with long-term oxygen therapy. J Telemed Telecare 2014;20(6):307-16. doi: 10.1177/1357633X14544421. Epub 2014 Jul 22 16. Imaz I, Rubio B, Cornejo AM, Gonzalez-Enriquez J. Budget impact and cost-utility analysis of universal infant rotavirus vaccination in Spain. Prev Med 2014 Apr;61:116-21. doi: 10.1016/j.ypmed.2013.12.013. Epub 2013 Dec 18. 17. González-Parra E, Gros B, Galán A, Herrero J, Oyagüez I, Keith M, et al. Análisis coste-efectividad de carbonato de lantano frente a clorhidrato de sevelámero en el tratamiento de la hiperfosfatemia en pacientes con enfermedad renal crónica en España. Pharmacoeconomics Spanish Research Articles 2014:1-12. 18. Gomez-Outes A, Avendano-Sola C, Terleira-Fernandez AI, Vargas-Castrillon E. Pharmacoeconomic Evaluation of Dabigatran, Rivaroxaban and Apixaban versus Enoxaparin for the Prevention of Venous Thromboembolism After Total Hip or Knee Replacement in Spain. Pharmacoeconomics 2014 Sep;32(9):919-36. doi: 10.1007/s40273-014-0175-5. 19. Gomez Leon N, Escalona S, Bandres B, Belda C, Callejo D, Blasco JA. F-Fluorodeoxyglucose Positron Emission Tomography/Computed Tomography Accuracy in the Staging of Non-Small Cell Lung Cancer: Review and Cost-Effectiveness. Radiol Res Pract 2014;2014:135934. 20. Dilla T, Moller J, P OD, Alvarez M, Sacristan JA, Happich M, et al. Long-acting olanzapine versus long-acting risperidone for schizophrenia in Spain inverted question mark a cost-effectiveness comparison. BMC Psychiatry 2014;14(1):298. 21. Collados C, Martín V, González-Torralba F, Rejas J. Análisis coste-efectividad de beclometasona/formoterol frente a fluticasona/salmeterol en el tratamiento de pacientes con asma moderada a grave en España. Pharmacoeconomics Spanish Research Articles 2014:1-10. 22. Coleman CI, Limone BL. Universal versus platelet reactivity assay- driven use of P2Y12 inhibitors in acute coronary syndrome patients. Cost-effectiveness analyses for six European perspectives. Thromb Haemost 2014;111(1). 23. Castilla I, Mar J, Valcarcel-Nazco C, Arrospide A, Ramos-Goni JM. Cost-Utility Analysis of Gastric Bypass for Severely Obese Patients in Spain. Obes Surg 2014 Dec;24(12):2061-8. doi: 10.1007/s11695-014-1304-0. 24. Capel M, Tornero J, Zamorano JL, Oyaguez I, Casado MA, Sanchez-Covisa J, et al. Efficiency of naproxen/esomeprazole in association for osteoarthrosis treatment in Spain. Reumatol Clin 2014. Jul-Aug;10(4):210-7. doi: 10.1016/j.reuma.2013.11.009. Epub 2013 Dec 29. 25. Buti M, Gros B, Oyaguez I, Andrade RJ, Serra MA, Turnes J, et al. [Cost-utility analysis of triple therapy with telaprevir in treatment-naive hepatitis C patients.]. Farm Hosp 2014;38(n05):418-29. 26. Brosa M, Garcia Del Muro X, Mora J, Villacampa A, Pozo-Rubio T, Cubells L, et al. Orphan drugs revisited: cost-effectiveness analysis of the addition of mifamurtide to the conventional treatment of osteosarcoma. Expert Rev Pharmacoecon Outcomes Res 2014:1-10. 27. Benejam-Gual JM, Sanz-Granda A, Garcia-Miralles Gravalos R, Severa-Ruiz de Velasco A, Pons-Viver J. Cost-effectiveness analysis at 2 years of surgical treatment of benign prostatic hyperplasia by photoselective vaporization of the prostate with GreenLight-Photo vaporization 120 W versus transurethral resection of the prostate. Actas Urol Esp 2014;38(4):238-43. 28. Barón Esquivias G, Escolar Albaladejo G, Zamorano JL, Betegón Nicolás L, Canal Fontcuberta C, de Salas-Cansado M, et al. Análisis coste-efectividad de apixabán frente a acenocumarol en la prevención del ictus en pacientes con fibrilación auricular no valvular en España. Rev Esp Cardiol 2014 Dec 9. pii: S1885-5857(14)00408-3. doi: 10.1016/j.rec.2014.08.010. [Epub ahead of print] 29. Aragones E, Lopez-Cortacans G, Sanchez-Iriso E, Pinol JL, Caballero A, Salvador-Carulla L, et al. Cost-effectiveness analysis of a collaborative care programme for depression in primary care. J Affect Disord 2014;159:85-93. 30. Angulo JC, Valpas A, Rejas J, Linden K, Kvasz M, Snedecor SJ. Cost Effectiveness of Fesoterodine and Tolterodine for the Treatment of Overactive Bladder with Urge Urinary Incontinence in Spain and Finland. Clin Drug Investig 2014 May;34(5):297-307. doi: 10.1007/s40261-014-0178-1. 31. Abad Paniagua EJ, Casado Escribano P, Fernandez Rodriguez JM, Morales Escobar FJ, Betegon Nicolas L, Sanchez-Covisa J, et al. [Cost-effectiveness analysis of dapagliflozin compared to DPP4 inhibitors and other oral antidiabetic drugs in the treatment of type-2 diabetes mellitus in Spain.]. Aten Primaria 2014 Dec 30. pii: S0212-6567(14)00390-4. doi: 10.1016/j.aprim.2014.11.002. [Epub ahead of print] 32. Villa G, Morano R, Roman A, Gil J. [Efficiency of initiation with ambrisentan versus bosentan in the treatment of pulmonary arterial hypertension.]. Farm Hosp 2013;37(5):358-65. 33. Vallejo-Torres L, Castilla Rodríguez I, Cuellar-Pompa L, Couce Pico ML, Pérez-Cerdá C, Martín Hernández E, et al. Análisis coste-efectividad del cribado neonatal de la deficiencia de biotinidasa: Red Española de Agencias de Evaluación de Tecnologías y Prestaciones del SNS del Ministerio de Sanidad, Servicios Sociales e Igualdad. Servicio de Evaluación del Servicio Canario de Salud, 2013. 34. Turnes J, Romero-Gomez M, Planas R, Sola R, Garcia-Samaniego J, Diago M, et al. Pharmacoeconomic analysis of the treatment of chronic hepatitis C with peginterferon alfa-2a or peginterferon alfa-2b plus ribavirin in Spain. Gastroenterol Hepatol 2013 Nov;36(9):555-64. doi: 10.1016/j.gastrohep.2013.08.003. Epub 2013 Oct 9. 35. Suárez-Fernández C, Gómez Cerezo JF, Gómez Arrayás I, Betegón Nicolás L, de Salas-Cansado M, Rubio Terrés C. Análisis de coste-efectividad de apixaban frente a enoxaparina en la prevención del tromboembolismo venoso en la artroplastia total de rodilla o cadera. Pharmacoeconomics Spanish Research Articles 2013;99(1). 36. Rubio-Valera M, Bosmans J, Fernandez A, Penarrubia-Maria M, March M, Trave P, et al. Cost-Effectiveness of a Community Pharmacist Intervention in Patients with Depression: A Randomized Controlled Trial (PRODEFAR Study). PLoS One 2013;8(8):e70588. 37. Ramirez de Arellano A, Coca A, de la Figuera M, Rubio-Terres C, Rubio-Rodriguez D, Gracia A, et al. Economic Evaluation of Cardio inCode, a Clinical-Genetic Function for Coronary Heart Disease Risk Assessment. Appl Health Econ Health Policy 2013 Oct;11(5):531-42 38. Parrondo J, Grande C, Ibanez J, Palau J, Paramo JA, Villa G. [Economic evaluation of Thrombopoietin Receptor Agonists in the treatment of chronic Primary Immune Thrombocytopenia.]. Farm Hosp 2013;37(3):182-91. 39. Paggiaro P, Patel S, Nicolini G, Pradelli L, Zaniolo O, Papi A. Stepping down from high dose fluticasone/salmeterol to extrafine BDP/F in asthma is cost-effective. Respir Med 2013;107(10):1531-7. 40. Montesino-Semper MF, Jimenez-Calvo JM, Cabases JM, Sanchez-Iriso E, Hualde-Alfaro A, Garcia-Garcia D. Cost-effectiveness analysis of the surgical treatment of female urinary incontinence using slings and meshes. Eur J Obstet Gynecol Reprod Biol 2013 Nov;171(1):180-6. doi: 10.1016/j.ejogrb.2013.08.035. Epub 2013 Aug 30. 41. Monreal M, Folkerts K, Diamantopoulos A, Imberti D, Brosa M. Cost-effectiveness impact of rivaroxaban versus new and existing prophylaxis for the prevention of venous thromboembolism after total hip or knee replacement surgery in France, Italy and Spain. Thromb Haemost 2013;110(5). 42. Mezquita Raya P, Perez A, Ramirez de Arellano A, Briones T, Hunt B, Valentine WJ. Incretin Therapy for Type 2 Diabetes in Spain: A Cost-Effectiveness Analysis of Liraglutide Versus Sitagliptin. Diabetes Ther 2013 Dec;4(2):417-30. doi: 10.1007/s13300-013-0044-9. Epub 2013 Oct 17. 43. Martino J, Gomez E, Bilbao JL, Duenas JC, Vazquez-Barquero A. Cost-utility of maximal safe resection of WHO grade II gliomas within eloquent areas. Acta Neurochir (Wien) 2013 Jan;155(1):41-50. doi: 10.1007/s00701-012-1541-8. 44. Luciano JV, Sabes-Figuera R, Cardenosa E, M TP-M, Fernandez-Vergel R, Garcia-Campayo J, et al. Cost-Utility of a Psychoeducational Intervention in Fibromyalgia Patients Compared With Usual Care: An Economic Evaluation Alongside a 12-Month Randomized Controlled Trial. Clin J Pain 2013. Aug;29(8):702-11. doi: 10.1097/AJP.0b013e318270f99a. 45. Lindner L, Vieta A, Rodríguez C, Barnadas A, Sánchez-Rovira P, Martín I. Análisis coste-efectividad de la terapia adyuvante con trastuzumab para el tratamiento de cáncer de mama en estadios iniciales. Pharmacoeconomics Spanish Research Articles 2013;99(1). 46. Hidalgo-Vega A, Ramos-Goni JM, Villoro R. Cost-utility of ranolazine for the symptomatic treatment of patients with chronic angina pectoris in Spain. Eur J Health Econ 2013. 10.1007/s10198-013-0534-8 47. Guirado Perich L, Fernández-Rivera C, Torre-Cisneros J, Sabater-Cabrera E, Ruiz-Beato E, Varela Moreno C. Análisis coste-utilidad de valganciclovir durante 200 días frente a 100 días post-trasplante como tratamiento preventivo de la enfermedad por citomegalovirus en receptores de trasplante renal de alto riesgo. Pharmacoeconomics Spanish Research Articles 2013;99(1). 48. Guest JF, Panca M, Sladkevicius E, Gough N, Linch M. Cost Effectiveness of First-Line Treatment with Doxorubicin/Ifosfamide Compared to Trabectedin Monotherapy in the Management of Advanced Soft Tissue Sarcoma in Italy, Spain, and Sweden. Sarcoma 2013;2013:725305. 49. Fonseca T, Clegg J, Caputo G, Norrbacka K, Dilla T, Alvarez M. The cost-effectiveness of exenatide once weekly compared with exenatide twice daily and insulin glargine for the treatment of patients with type two diabetes and body mass index >/= 30 kg/m in Spain. J Med Econ 2013. Jul;16(7):926-38. doi: 10.3111/13696998.2013.803110. Epub 2013 May 31. 50. Ferreira-González I, Serra V, Abdul O, Paz S, Lizan L, Banz K, et al. Evidencia del coste-efectividad de la implantación transcatéter de la prótesis valvular aórtica (TAVI) Edwards SAPIEN en pacientes de alto riesgo con estenosis aórtica sintomática en España: resultados preliminares. Pharmacoeconomics Spanish Research Articles 2013;10(1). 51. Dembek C, White LA, Quach J, Szkurhan A, Rashid N, Blasco MR. Cost-effectiveness of injectable disease-modifying therapies for the treatment of relapsing forms of multiple sclerosis in Spain. Eur J Health Econ 2013. doi: 10.1007/s10198-013-0478-z. Epub 2013 Apr 25. 52. De Salas-Cansado M, Alvarez E, Olivares JM, Carrasco JL, Ferro MB, Rejas J. Modelling the cost-effectiveness of pregabalin versus usual care in daily practice in the treatment of refractory generalised anxiety disorder in Spain. Soc Psychiatry Psychiatr Epidemiol 2013;48(6):985-96. doi: 10.1007/s00127-012-0606-6. Epub 2012 Oct 21 53. Davies A, Sculpher M, Barrett A, Huete T, Sacristan JA, Dilla T. Prasugrel compared to clopidogrel in patients with acute coronary syndrome undergoing percutenaous coronary intervention: a Spanish model-based cost effectiveness analysis. Farm Hosp 2013;37(4):307-16. 54. Darba J, Perez-Alvarez N, Kaskens L, Holgado-Perez S, Racketa J, Rejas J. Cost-effectiveness of bazedoxifene versus raloxifene in the treatment of postmenopausal women in Spain. Clinicoecon Outcomes Res 2013;5:327-36. 55. Crespo C, Moreno E, Sierra J, Serip S, Rubio M. Cost-effectiveness analysis of azacitidine in the treatment of high-risk myelodysplastic syndromes in Spain. Health Econ Rev 2013;3(1):28. 56. Castilla Rodríguez I, Valcárcel Nazco C, Vallejo-Torres L, Cela E, Posada M, Dulín-Íñiguez E, et al. Análisis coste-efectividad del cribado neonatal de la enfermedad de células falciformes. Red Española de Agencias de Evaluación de Tecnologías y Prestaciones del SNS del Ministerio de Sanidad, Servicios Sociales e Igualdad. Servicio de Evaluación del Servicio Canario de Salud, 2013. 57. Carrion JA, Gonzalez-Colominas E, Garcia-Retortillo M, Canete N, Cirera I, Coll S, et al. A multidisciplinary support programme increases the efficiency of pegylated interferon alfa-2a and ribavirin in hepatitis C. J Hepatol 2013;59(5):926-33. doi: 10.1016/j.jhep.2013.06.019. Epub 2013 Jun 26. 58. Calvo M, Subirats L, Ceccaroni L, Maroto JM, de Pablo C, Miralles F. Automatic assessment of socioeconomic impact on cardiac rehabilitation. Int J Environ Res Public Health 2013;10(11):5266-83. 59. Brosa Riestra M, Pérez-Alcántara F, Borderías Clau L, Galdiz-Iturri JB, Riera-Febrer M, Figueras Sabaté M. Análisis de coste-utilidad de indacaterol frente a tiotropio en el tratamiento de la EPOC en España. Pharmacoeconomics Spanish Research Articles 2013;99(1). 60. Blazquez-Perez A, San Miguel R, Mar J. Cost-Effectiveness Analysis of Triple Therapy with Protease Inhibitors in Treatment-Naive Hepatitis C Patients. Pharmacoeconomics 2013 Oct;31(10):919-31. doi: 10.1007/s40273-013-0080-3. 61. Almenar L, Diaz B, Quesada A, Crespo C, Marti B, Mealing S, et al. Cost-Effectiveness Analysis of Cardiac Resynchronization Therapy in Patients with Nyha I and Nyha Ii Heart Failure in Spain. Int J Technol Assess Health Care 2013:1-7. 62. Alba E, Ciruelos E, Lopez R, Lopez-Vega JM, Lluch A, Martin M, et al. Cost-utility analysis of nanoparticle albumin-bound paclitaxel versus paclitaxel in monotherapy in pretreated metastatic breast cancer in Spain. Expert Rev Pharmacoecon Outcomes Res 2013 Jun;13(3):381-91. doi: 10.1586/erp.13.18. Epub 2013 Mar 28. 63. Wilson K, Hettle R, Marbaix S, Cerezo SD, Ines M, Santoni L, et al. An economic evaluation based on a randomized placebo-controlled trial of varenicline in smokers with cardiovascular disease: results for Belgium, Spain, Portugal, and Italy. Eur J Cardiovasc Prev Rehabil 2012 Oct;19(5):1173-83. doi: 10.1177/1741826711420345. Epub 2011 Aug 12. 64. Villa G, Sanchez-Alvarez E, Cuervo J, Fernandez-Ortiz L, Rebollo P, Ortega F. Cost-effectiveness analysis of timely dialysis referral after renal transplant failure in Spain. BMC Health Serv Res 2012;12(1):257. 65. Villa G, Fernandez-Ortiz L, Cuervo J, Rebollo P, Selgas R, Gonzalez T, et al. Cost-Effectiveness Analysis of the Spanish Renal Replacement Therapy Program. Perit Dial Int 2012;32(2):192-9. 66. Trujillo Martí M, Rodríguez Rodríguez L. Efectividad, seguridad y coste-efectividad del ipilimumab en el tratamiento del melanoma avanzado. Servicio de Evaluación del Servicio Canario de Salud, 2012. 67. Treur M, Baca E, Bobes J, Canas F, Salvador L, Gonzalez B, et al. The cost-effectiveness of paliperidone extended release in Spain. J Med Econ 2012; Suppl 1:26-34. doi: 10.3111/13696998.2012.734884. Epub 2012 Oct 23. 68. Slof J, Gras A. Sativex((R)) in multiple sclerosis spasticity: a cost-effectiveness model. Expert Rev Pharmacoecon Outcomes Res 2012 Aug;12(4):439-41. doi: 10.1586/erp.12.40. Epub 2012 Jun 8 69. Sanchez-de la Rosa R, Sabater E, Casado M, Arroyo R. Cost-effectiveness Analysis of Disease Modifiying Drugs (Interferons and Glatiramer Acetate) as first line treatments in Remitting-Relapsing Multiple Sclerosis patients. J Med Econ 2012;15(3):424-33. doi: 10.3111/13696998.2012.654868. Epub 2012 Jan 27. 70. Rubio-Rodriguez D. [Economic analysis of specialised and intensive promotion of breastfeeding in neonatal units in Spain.]. An Pediatr (Barc) 2012;77(5):297-308. doi: 10.1016/j.anpedi.2012.03.015. Epub 2012 May 9. 71. Roman A, Barbera JA, Escribano P, Sala ML, Febrer L, Oyaguez I, et al. Cost Effectiveness of Prostacyclins in Pulmonary Arterial Hypertension. Appl Health Econ Health Policy 2012 May 1;10(3):175-88. doi: 10.2165/11630780-000000000-00000. 72. Quintana Díaz M, Borobia AM, Pérez Cachafeiro S, Rodríguez C, García Erce JA. Estudio de coste-efectividad del empleo de concentrado de complejo protrombínico en urgencias para evitar las complicaciones de la sobredosificación de anticoagulantes. Emergencias 2012;24:113-20. 73. Pueyo MJ, Larrosa M, Suris X, Garcia-Ruiz AJ. Cost-utility and budget impact analysis of primary prevention with alendronate of osteoporotic hip fractures in Catalonia. Reumatol Clin 2012 May-Jun;8(3):128-34. doi: 10.1016/j.reuma.2012.01.001. Epub 2012 Mar 14 74. Obradovic M, Ikenberg R, Hertel N, Antonanzas F, Galvez R, Liedgens H. Cost-Effectiveness of Tapentadol in Severe Chronic Pain in Spain: A Cost Analysis of Data From RCTs. Clin Ther 2012. 75. Montalar J, Cajaraville G, Carreras MJ, Rubio MJ, García-San Andrés B, Oyagüez I, et al. Trabectedin plus PLD versus PLD monotherapy in patients with platinum-sensitive relapsed ovarian cancer: a cost-effectiveness analysis in Spain. Eur J Hosp Pharm 2012;19:364-69. 76. Lindner L, Ancochea J, Aparicio J, Camíns L. Coste-efectividad de roflumilast en el tratamiento de pacientes con enfermedad pulmonar obstructiva crónica en España. Pharmacoeconomics - Spanish Research Articles 2012;99(1). 77. Iannazzo S, Carsi M, Chiroli S. A Cost-Utility Analysis of Cinacalcet in Secondary Hyperparathyroidism in Five European Countries. Appl Health Econ Health Policy 2012 Mar 1;10(2):127-38. doi: 10.2165/11597980-000000000-00000. 78. Gonzalez-Juanatey JR, Alvarez-Sabin J, Lobos JM, Martinez-Rubio A, Reverter JC, Oyaguez I, et al. Cost-effectiveness of Dabigatran for Stroke Prevention in Non-valvular Atrial Fibrillation in Spain. Rev Esp Cardiol. 2012 Oct;65(10):901-10. doi: 10.1016/j.recesp.2012.06.006. Epub 2012 Sep 6. 79. Gómez Cerezo JF, Gómez Arrayas I, Suárez Fernández C, Betegon L, de Salas-Cansado M, Rubio Terrés C. Análisis coste-efectividad de apixaban frente a dabigatrán en la prevención de la tromboembolia venosa en pacientes intervenidos de artroplastia total de rodilla o de cadera. Rev Esp Cir Ortop Traumatol 2012;56:459-70. 80. Garcia-Ruiz AJ, Perez-Costillas L, Montesinos AC, Alcalde J, Oyaguez I, Casado MA. Cost-effectiveness analysis of antipsychotics in reducing schizophrenia relapses. Health Econ Rev 2012;2(1):8. 81. García Ruíz AJ, Divisón Garrote JA, García-Agua Soler N, Morata García de la Puerta F, Montesinos AC, Ávila Lachica L. Análisis coste-eficacia de los antihipertensivos en dosis fijas. Semergen 2012. doi: 10.1016/j.semerg.2012.05.011. Epub 2012 Sep 5. 82. Gálvez R, Provencio M, Sanz-Ortíz J, Ribera M, Esteban E, Collado F, et al. Análisis económico del oxicodona LP/naloxona LP en el manejo del dolor intenso y el estreñimiento asociado al tratamiento con opioides en España. Pharmacoeconomics - Spanish Research Articles 2012. 83. Fernandez-Fairen M, Murcia A, Torres A, Hernandez-Vaquero D, Menzie AM. Is anterior cervical fusion with a porous tantalum implant a cost-effective method to treat cervical disc disease with radiculopathy? Spine (Phila Pa 1976) 2012;37(20):1734-41. 84. de Salas-Cansado M, Perez C, Saldana MT, Navarro A, Rejas J. A cost-effectiveness analysis of the effect of pregabalin versus usual care in the treatment of refractory neuropathic pain in routine medical practice in Spain. Pain Med 2012;13(5):699-710. 85. de Salas-Cansado M, Perez C, Saldana MT, Navarro A, Gonzalez-Gomez FJ, Ruiz L, et al. An economic evaluation of pregabalin versus usual care in the management of community-treated patients with refractory painful diabetic peripheral neuropathy in primary care settings. Prim Care Diabetes 2012 Dec;6(4):303-12. doi: 10.1016/j.pcd.2012.03.001. Epub 2012 May 15. 86. De Salas-Cansado M, Olivares JM, Alvarez E, Carrasco JL, Barrueta A, Rejas J. Pregabalin versus SSRIs and SNRIs in benzodiazepine-refractory outpatients with generalized anxiety disorder: a post hoc cost-effectiveness analysis in usual medical practice in Spain. Clinicoecon Outcomes Res 2012;4:157-68. 87. Darbá J, Kaskens L. Análisis Coste-Efectividad de Dasatinib frente a Imatinib a dosis alta en el tratamiento de la leucemia mieloide crónica en pacientes resistentes a dosis estándar de imatinib. Pharmacoeconomics Spanish Research Articles 2012;9(2). 88. Crespo C, Perez-Simon JA, Rodriguez JM, Sierra J, Brosa M. Development of a Population-Based Cost-Effectiveness Model of Chronic Graft-Versus-Host Disease in Spain. Clin Ther 2012;34(8):1774-87. doi: 10.1016/j.clinthera.2012.06.029. Epub 2012 Jul 25. 89. Castro Gomez AJ, Lopez-Guillermo A, Rueda Dominguez A, Salar A, Varela Moreno C, Rubio-Terres C. [Cost-effectiveness analysis of maintenance therapy with rituximab in patients with follicular lymphoma responding to induction therapy at the first line]. Rev Esp Salud Publica 2012;86(2):163-76. 90. Castilla Rodríguez I, Cuéllar-Pompa L, García Pérez L, Perestelo-Pérez L, Pinilla Domínguez P, Ramos Goñi JM, et al. Análisis coste-utilidad de la asenapina versus olanzapina en el tratamiento del trastorno bipolar tipo I. Informes de Evaluación de Tecnologías Sanitarias. Santa Cruz de Tenerife: Servicio de Evaluación del Servicio Canario de la Salud, 2012. 91. Casado Buesa MI, García Hernández L, González Enríquez J, Imaz Iglesia I, Rubio González B, Zegarra Salas P. Evaluación económica de la introducción de la vacuna contra VPH en España para la prevención del cáncer de cuello uterino. Informes de Evaluación de Tecnologías Sanitarias (IPE 2012/69). Madrid: Agencia de Evaluación de Tecnologías Sanitarias, 2012. 92. Brosa Riestra M, Monreal M, Piñol C. Análisis coste-efectividad de rivaroxabán en la prevención de la enfermedad tromboembólica venosa en España. Pharmacoeconomics - Spanish Research Articles 2012;9(1). 93. Bayón Yusta JC, Orruño Aguado E, Asua Batarrita J, Artetxe Ocasar J, Emparanza Knör JI, Gorostiza Hormaetxe I. Evaluación de los resultados de la telemonitorización de pacientes con insuficiencia cardiaca y enfermedad obstructiva crónica. Informes de Evaluación de Tecnologías Sanitarias. Vitoria-Gasteiz: Agencia de Evaluación de Tecnologías Sanitarias del País Vasco, 2012. 94. Barrios V, Lobos JM, Serrano A, Brosa M, Capel M, Alvarez Sanz C. Cost-effectiveness analysis of rosuvastatin versus generic atorvastatin in Spain. J Med Econ 2012;15 Suppl 1:45-54. doi: 10.3111/13696998.2012.726674. Epub 2012 Sep 12. 95. Sicuri E, Munoz J, Pinazo MJ, Posada E, Sanchez J, Alonso PL, et al. Economic evaluation of Chagas disease screening of pregnant Latin American women and of their infants in a non endemic area. Acta Trop 2011;118(2):110-7. 96. Perez-Rubio A, Luquero FJ, Bouza JM, Castrodeza Sanz JJ, Bachiller Luque MR, de Lejarazu RO, et al. Socio-economic modelling of rotavirus vaccination in Castilla y Leon, Spain. Infez Med 2011;19(3):166-75. 97. Oyagüez I, Gómez Alonso C, Marqués De Torrés M, García Coscolín T, Betegon L, Casado M. Evaluación de la eficiencia de risedronato 75 mg frente a alendronato genérico 70 mg, en mujeres con osteoporosis postmenopáusica y fractura vertebral previa en España. Rev Osteoporos Metab Miner 2011;3(1):21-29. 98. Morano R, Perez F, Brosa M, Escolano IP. [Cost-effectiveness analysis of pneumococcal vaccination in Spain]. Gac Sanit 2011;25(4):267-73. 99. López-Sendón JL, Martí-Sánchez D, Martí B, Oyagüez I, Casado MÁ. Cost-effective analysis of eplerenone in patients with post-acute myocardial infarction heart failure. Pharmacoeconomics - Spanish Research Articles 2011;8(2):39-50. 100. Lock K, Wilson K, Murphy D, Riesco JA. A cost-effectiveness model of smoking cessation based on a randomised controlled trial of varenicline versus placebo in patients with chronic obstructive pulmonary disease. Expert Opin Pharmacother 2011 Dec;12(17):2613-26. doi: 10.1517/14656566.2011.628935. Epub 2011 Oct 21. 101. Iannazzo S, Carsi M, Sorio F, Chiroli S. Análisis coste-utilidad de cinacalcet en el hiperparatiroidismo secundario en España. Pharmacoeconomics - Spanish Research Articles 2011. 102. Goodall G, Costi M, Timlin L, Reviriego J, Sacristan JA, Smith-Palmer J, et al. Cost-effectiveness of exenatide versus insulin glargine in Spanish patients with obesity and type 2 diabetes mellitus. Endocrinol Nutr 2011 Aug-Sep;58(7):331-40. doi: 10.1016/j.endonu.2011.04.005. Epub 2011 Jun 29. 103. Felipe Casado L, Garcia Marco JA, Gilsanz F, Gonzalez M, Rios E, de la Serna J, et al. [Economic evaluation of rituximab added to fludarabine plus cyclophosphamide versus fludarabine plus cyclophosphamide for the treatment of chronic lymphocytic leukemia]. Gac Sanit 2011;25(4):274-81. 104. Diez-Domingo J, Ridao-Lopez M, Gutierrez-Gimeno MV, Puig-Barbera J, Lluch-Rodrigo JA, Pastor-Villalba E. Pharmacoeconomic assessment of implementing a universal PCV-13 vaccination programme in the Valencian public health system (Spain). Vaccine 2011 Dec 6;29(52):9640-8. doi: 10.1016/j.vaccine.2011.10.038. Epub 2011 Oct 24. 105. Carles M, Vilaprinyo E, Cots F, Gregori A, Pla R, Roman R, et al. Cost-effectiveness of early detection of breast cancer in Catalonia (Spain). BMC Cancer 2011;11:192. 106. Calvo Aller E, Maroto P, Kreif N, Gonzalez Larriba JL, Lopez-Brea M, Castellano D, et al. Cost-effectiveness evaluation of sunitinib as first-line targeted therapy for metastatic renal cell carcinoma in Spain. Clin Transl Oncol 2011;13(12):869-77. 107. Borgstrom F, Strom O, Kleman M, McCloskey E, Johansson H, Oden A, et al. Cost-effectiveness of bazedoxifene incorporating the FRAX(R) algorithm in a European perspective. Osteoporos Int 2011;22(3):955-65. 108. Arlandis-Guzman S, Errando-Smet C, Trocio J, Arumi D, Rejas J. Cost-effectiveness analysis of antimuscarinics in the treatment of patients with overactive bladder in Spain: A decision-tree model. BMC Urol 2011;11:9. 109. Arlandis S, Castro D, Errando C, Fernandez E, Jimenez M, Gonzalez P, et al. Cost-effectiveness of sacral neuromodulation compared to botulinum neurotoxin a or continued medical management in refractory overactive bladder. Value Health 2011;14(2):219-28. 110. Antonanzas F, Brenes F, Molero JM, Fernandez-Pro A, Huerta A, Palencia R, et al. [Cost-effectiveness of the combination therapy of dutasteride and tamsulosin in the treatment of benign prostatic hyperlasia in Spain]. Actas Urol Esp 2011;35(2):65-71. 111. Vera-Llonch M, Dukes E, Rejas J, Sofrygin O, Mychaskiw M, Oster G. Cost-effectiveness of pregabalin versus venlafaxine in the treatment of generalized anxiety disorder: findings from a Spanish perspective. Eur J Health Econ 2010;11(1):35-44. 112. Tunis SL, Willis WD, Foos V. Self-monitoring of blood glucose (SMBG) in patients with type 2 diabetes on oral anti-diabetes drugs: cost-effectiveness in France, Germany, Italy, and Spain. Curr Med Res Opin 2010;26(1):163-75. 113. Ramos Goñi JM, Mar J. Coste-Efectividad del tratamiento endovascular frente a cirugía abierta en pacientes con enfermedad esteno-oclusiva de la arteria femoral. Informes de Evaluación de Tecnologías Sanitarias. SESCS 2007/01. Santa Cruz de Tenerife: Servicio de Evaluación del Servicio Canario de la Salud, 2010. 114. Ramos Goñi JM, Baldi S, Valles González H, Simonetti G, Buceta E, Maynar M. Coste-efectividad de la colocación aislada de stent frente al stent con angioplastia por balón y protección vascular en la arteriosclerosis carotidea. . Informes de Evaluación de Tecnologías Sanitarias: SESCS Nº 2007/17. Santa Cruz de Tenerife: Servicio de Evaluación del Servicio Canario de la Salud, 2010. 115. Picazo J, Méndez C, Oyagüez I, Casado MA, Guijarro P. Coste-utilidad de la incorporación de las nuevas vacunas antineumocócicas conjugadas al programa de vacunación de la comunidad de Madrid. Impacto sobre la enfermedad neumocócica invasora. Vacunas 2010;11(3):96-104. 116. Paz-Ares L, del Muro JG, Grande E, Diaz S. A cost-effectiveness analysis of sunitinib in patients with metastatic renal cell carcinoma intolerant to or experiencing disease progression on immunotherapy: perspective of the Spanish National Health System. J Clin Pharm Ther 2010;35(4):429-38. 117. Nuijten MJ, Wittenberg W. Cost effectiveness of palivizumab in Spain: an analysis using observational data. Eur J Health Econ 2010;11(1):105-15. 118. Muñoz R, Martínez-Ferrer J, Delgado J, Rodríguez Barrios JM, Caro JJ, Guo S. Can adding cardiac resynchronization compensate to optimized pharmacological treatment in patients with heart failure in Spain? Pharmacoeconomics - Spanish Research Articles 2010;7(1):13-25. 119. Moreno S, Gonzalez J, Lekander I, Marti B, Oyaguez I, Sanchez-de la Rosa R, et al. Cost-effectiveness of optimized background therapy plus maraviroc for previously treated patients with R5 HIV-1 infection from the perspective of the Spanish health care system. Clin Ther 2010;32(13):2232-45. 120. Lopez-Bastida J, Sassi F, Bellas Beceiro B, Garcia Pérez L. Análisis coste-efectividad del cribado del cáncer colorectal en la población general. Informes de Evaluación de Tecnologías Sanitarias: SESCS Nº 2007/17. Santa Cruz de Tenerife: Servicio de Evaluación del Servicio Canario de la Salud, 2010. 121. Lopez Gude MJ, Rodriguez Bezos D, Rodriguez Barrios JM. [Cost-benefit analysis of concomitant atrial fibrillation management in Spain]. Gac Sanit 2010;24(1):59-65. 122. Imaz Iglesia I, Rubio González B, López Delgado ME, Amate Blanco JM, Gómez Pajuelo P, González Enríquez J. Análisis coste-utilidad de los tratamientos farmacológicos para la prevención de fracturas en mujeres con osteoporosis en España. Informe público de Evaluación de Tecnologías Sanitarias IPE 63/2010. Madrid: Agencia de Evaluación de Tecnologías Sanitarias, Instituto de Salud Carlos III, 2010. 123. Hernandez-Pastor LJ, Ortega A, Garcia-Layana A, Giraldez J. Cost-effectiveness of ranibizumab compared with pegaptanib in neovascular age-related macular degeneration. Graefes Arch Clin Exp Ophthalmol 2010;248(4):467-76. 124. Gómez J, Ríos E, Rubio C, Castro Gómez A, Varela C. Pharmacoeconomic analysis of adding rituximab to chemotherapy as first line treatment for patients with advanced follicular lymphoma. Pharmacoeconomics - Spanish Research Articles 2010;7(2):55-67. 125. Frias C, Cortes J, Segui MA, Oyaguez I, Casado MA. Cost-effectiveness analyses of docetaxel versus paclitaxel once weekly in patients with metastatic breast cancer in progression following anthracycline chemotherapy, in Spain. Clin Transl Oncol 2010;12(10):692-700. 126. Crespo C, Brosa M, Galvan J, Carbonell J, Maymo J, Marenco JL, et al. [Pharmacoeconomic analysis of Metoject((R)) in the treatment of rheumatoid arthritis in Spain]. Reumatol Clin 2010;6(4):203-11. 127. Callejo D, López-Polín A, Guerra M, Blasco JA. Evaluación económica de la vaporización fotoselectiva de la próstata para el tratamiento de la hiperplasia benigna de próstata. Informes de Evaluación de Tecnologías Sanitarias: UETS 09/02. Madrid: Unidad de Evaluación de Tecnologías Sanitarias, Agencía Laín Entralgo, 2010. 128. Callejo D, Guerra M, Hernandez-Madrid A, Blasco JA. Economic assessment of cardiac resynchronization therapy. Rev Esp Cardiol 2010;63(11):1235-43. 129. Asukai Y, Valladares A, Camps C, Wood E, Taipale K, Arellano J, et al. Cost-effectiveness analysis of pemetrexed versus docetaxel in the second-line treatment of non-small cell lung cancer in Spain: results for the non-squamous histology population. BMC Cancer 2010;10:26. 130. Arrospide A, Mar J, Vivancos-Mora J, Rejas-Gutierrez J, Caro J. [Cost-effectiveness analysis of using high doses of atorvastatin for the secondary stroke prevention in Spain]. Rev Neurol 2010;51(1):1-11. 131. Taylor DC, Pandya A, Thompson D, Chu P, Graff J, Shepherd J, et al. Cost-effectiveness of intensive atorvastatin therapy in secondary cardiovascular prevention in the United Kingdom, Spain, and Germany, based on the Treating to New Targets study. Eur J Health Econ 2009;10(3):255-65. 132. Serrano-Blanco A, Suarez D, Pinto-Meza A, Penarrubia MT, Haro JM. Fluoxetine and imipramine: are there differences in cost-utility for depression in primary care? J Eval Clin Pract 2009;15(1):195-203. 133. Schwander B, Gradl B, Zollner Y, Lindgren P, Diener HC, Luders S, et al. Cost-utility analysis of eprosartan compared to enalapril in primary prevention and nitrendipine in secondary prevention in Europe--the HEALTH model. Value Health 2009;12(6):857-71. 134. Rubio-Terrés C, Montejo González ÁL, Puchol Incertis M, Álvarez Sanz C. Pharmacoeconomic analysis of switching antipsychotic treatment, for inefficiency or adverse effects, to extended-release quetiapine. Pharmacoeconomics - Spanish Research Articles 2009;6(4):105-14. 135. Moreu J, Cequier A, Brosa M, Rodriguez JM, Crespo C, Hernandez JM, et al. [Economic evaluation and budget impact analysis of the Endeavor drug-eluting stent in Spain]. Gac Sanit 2009;23(6):540-7. 136. Martin-Jimenez M, Rodriguez-Lescure A, Ruiz-Borrego M, Segui-Palmer MA, Brosa-Riestra M. Cost-effectiveness analysis of docetaxel (Taxotere) vs. 5-fluorouracil in combined therapy in the initial phases of breast cancer. Clin Transl Oncol 2009;11(1):41-7. 137. Lopez-Bastida J, Hart W, Garcia-Perez L, Linertova R. Cost-effectiveness of donepezil in the treatment of mild or moderate Alzheimer's disease. J Alzheimers Dis 2009;16(2):399-407. 138. Latour-Perez J, de-Miguel-Balsa E. Cost effectiveness of fondaparinux in non-ST-elevation acute coronary syndrome. Pharmacoeconomics 2009;27(7):585-95. 139. Hong J, Dilla T, Arellano J. A modelled economic evaluation comparing atomoxetine with methylphenidate in the treatment of children with attention-deficit/hyperactivity disorder in Spain. BMC Psychiatry 2009;9:15. 140. Gschwend MH, Aagren M, Valentine WJ. Cost-effectiveness of insulin detemir compared with neutral protamine Hagedorn insulin in patients with type 1 diabetes using a basal-bolus regimen in five European countries. J Med Econ 2009;12(2):114-23. 141. Chaudhary MA, Moreno S, Kumar RN, Nocea G, Elbasha E. Cost-effectiveness analysis of raltegravir in treatment-experienced HIV type 1-infected patients in Spain. AIDS Res Hum Retroviruses 2009;25(7):679-89. 142. Cairols-Castellote MA, Salmerón-Febres LM, Fernández-Samos R, Iborra-Ortega E, Vaquero-Puerta C, Marco-Luque M, et al. Análisis coste-efectividad del tratamiento del aneurisma de aorta abdominal mediante prótesis endovascular en España. Angiología 2009;61(2):51-61. 143. Buti M, Brosa M, Casado MA, Rueda M, Esteban R. Modeling the cost-effectiveness of different oral antiviral therapies in patients with chronic hepatitis B. J Hepatol 2009;51(4):640-6. 144. Brosa M, De La Figuera M, Franch J, Segura J, Riera M, Figueras M. Cost-effective analysis of aliskiren in hypertensive patients with type 2 diabetes and nephropathy already treated with optimal angiotensin II receptor antagonists and other antihypertensive drugs. Pharmacoeconomics - Spanish Research Articles 2009;6(3):91-100. 145. Betegón L, Gómez C, Marqués De Torrés M. Análisis farmacoeconómico de risedronato semanal frente a alendronato semanal en españa*. Revista Española de Enfermedades Metabólicas Oseas 2009;18(1):9-14. 146. Anselmino M, Bammer T, Fernandez Cebrian JM, Daoud F, Romagnoli G, Torres A. Cost-effectiveness and budget impact of obesity surgery in patients with type 2 diabetes in three European countries(II). Obes Surg 2009;19(11):1542-9. 147. Schwarz B, Gouveia M, Chen J, Nocea G, Jameson K, Cook J, et al. Cost-effectiveness of sitagliptin-based treatment regimens in European patients with type 2 diabetes and haemoglobin A1c above target on metformin monotherapy. Diabetes Obes Metab 2008;10 Suppl 1:43-55. 148. Paz-Ares L, Garcia del Muro X, Grande E, Gonzalez P, Brosa M, Diaz S. Cost-effectiveness analysis of sunitinib in patients with metastatic and/or unresectable gastrointestinal stroma tumours (GIST) after progression or intolerance with imatinib. Clin Transl Oncol 2008;10(12):831-9. 149. Palmer JL, Goodall G, Nielsen S, Kotchie RW, Valentine WJ, Palmer AJ, et al. Cost-effectiveness of insulin aspart versus human soluble insulin in type 2 diabetes in four European countries: subgroup analyses from the PREDICTIVE study. Curr Med Res Opin 2008;24(5):1417-28. 150. Olry de Labry Lima A, Sordo del Castillo L, Garcia Mochon L, Epstein D, Bermudez Tamayo C, Villegas Portero R. [An economic assessment of genetic testing for familial adenomatous polyposis]. Rev Esp Enferm Dig 2008;100(8):470-5. 151. Munoz-Duyos A, Navarro-Luna A, Brosa M, Pando JA, Sitges-Serra A, Marco-Molina C. Clinical and cost effectiveness of sacral nerve stimulation for faecal incontinence. Br J Surg 2008;95(8):1037-43. 152. Mayordomo Cámara JI, López Pousa A, Jiménez García E, Badia X, Lara Surinyach N, Sorio Vilela F, et al. Evaluación Económica del uso de pegfilgrastim frente a filgrastim en profilaxis primaria en pacientes con cáncer de mama con riesgo de padecer neutropenia febril en España. Pharmacoeconomics - Spanish Research Articles 2008;5(3):71-81. 153. Martín Saborido C, Blasco Amaro JA, Callejo Velasco D, López-Pedraza Gómez MJ, Maeso Martínez S. Evaluación económica de los stents recubiertos de fármacos en el tratamiento de la cardiopatía isquémica. Informes de Evaluación de Tecnologías Sanitarias. Madrid: Unidad de Evaluación de Tecnologías Sanitarias, Agencia Laín Entralgo, 2008. 154. Lopez-Bastida J, Bellas Beceiro B, Garcia Pérez L. Análisis coste-efectividad del cribado del cáncer de mama mediante mamografía en diferentes grupos de edad (40 a 49, 50 a 69 y 70 a 75). Informes de Evaluación de Tecnologías Sanitarias: SESCS 2006/09. Madrid: Servicio de Evaluación del Servicio Canario de Salud, 2008. 155. Latour-Perez J, de Miguel Balsa E, Betegon L, Badia X. Using triple antiplatelet therapy in patients with non-ST elevation acute coronary syndrome managed invasively: a cost-effectiveness analysis. Value Health 2008;11(5):853-61. 156. Largeron N, Rémy V, Oyee J, San-Martín M, Cortés J, Olmos L. Análisis de coste-efectividad de la vacunación frente al virus del papiloma humano tipos 6, 11, 16 y 18 en España. Vacunas 2008;9(1):3-11. 157. Kobelt G, Sobocki P, Mulero J, Gratacos J, Collantes-Estevez E, Braun J. The cost-effectiveness of infliximab in the treatment of ankylosing spondylitis in Spain. Comparison of clinical trial and clinical practice data. Scand J Rheumatol 2008;37(1):62-71. 158. Hernandez-Pastor LJ, Ortega A, Garcia-Layana A, Giraldez J. Cost-effectiveness of ranibizumab compared with photodynamic treatment of neovascular age-related macular degeneration. Clin Ther 2008;30(12):2436-51. 159. Gusi N, Tomas-Carus P. Cost-utility of an 8-month aquatic training for women with fibromyalgia: a randomized controlled trial. Arthritis Res Ther 2008;10(1):R24. 160. Gusi N, Reyes MC, Gonzalez-Guerrero JL, Herrera E, Garcia JM. Cost-utility of a walking programme for moderately depressed, obese, or overweight elderly women in primary care: a randomised controlled trial. BMC Public Health 2008;8:231. 161. Folicular GdFdL. [Rituximab cost analysis for maintenance treatment of patients with follicular lymphoma]. Farm Hosp 2008;32(1):25-34. 162. Fernandez de Bobadilla Osorio J, Sanchez-Maestre C, Brosa Riestra M, Arroyo O, Sanz de Burgoa V, Wilson K. [Cost effectiveness analysis of varenicline (Champix) for the treatment of smoking in Spain]. An Med Interna 2008;25(7):342-8. 163. Cuadros Celorrio M, Villegas Portero R. HER2 en la selección de candidatas a tratamiento con Trastuzumab. Determinación del estado de HER2 en mujeres con cáncer de mama. Informes, Estudios e Investigación. Ministerio de Sanidad y Consumo. AETSA 2006/06. Sevilla: Agencia de Evaluación de Tecnologías Sanitarias de Andalucía, 2008. 164. Brosa M, Pérez V, Comas A, Segu JL. Análisis coste-efectividad de tres opciones terapéuticas iniciales en el tratamiento de la depresión mayor en España. Revista Española de Economía de la Salud 2008;7(4):165-72. 165. Brosa M, Munoz-Duyos A, Navarro-Luna A, Rodriguez JM, Serrano D, Gisbert R, et al. Cost-effectiveness analysis of sacral neuromodulation (SNM) with Interstim for fecal incontinence patients in Spain. Curr Med Res Opin 2008;24(3):907-18. 166. Brosa M, Buti M, Hay J, Staginnus U, Yuan Y, Zammit D. Coste-efectividad de entecavir en la hepatitis B crónica. Revista Española de Economía de la Salud 2008;7(3):111-18. 167. Ayuso-Mateos JL, Gutierrez-Recacha P, Haro JM, Salvador-Carulla L, Vázquez-Polo FJ, Negrín-Hernández MA, et al. Reducing the Burden of Mental Illness in Spain: population-level impact and cost-effectiveness of treatments in depression and schizophrenia. Informes Economía y Salud. Bilbao: Fundación BBVA, 2008. 168. Valldeoriola F, Morsi O, Tolosa E, Rumia J, Marti MJ, Martinez-Martin P. Prospective comparative study on cost-effectiveness of subthalamic stimulation and best medical treatment in advanced Parkinson's disease. Mov Disord 2007;22(15):2183-91. 169. Strom O, Borgstrom F, Sen SS, Boonen S, Haentjens P, Johnell O, et al. Cost-effectiveness of alendronate in the treatment of postmenopausal women in 9 European countries--an economic evaluation based on the fracture intervention trial. Osteoporos Int 2007;18(8):1047-61. 170. Simpson KN, Jones WJ, Rajagopalan R, Dietz B. Cost effectiveness of lopinavir/ritonavir tablets compared with atazanavir plus ritonavir in antiretroviral-experienced patients in the UK, France, Italy and Spain. Clin Drug Investig 2007;27(12):807-17. 171. Sanchez JL, Pereperez SB, Bastida JL, Martinez MM. Cost-utility analysis applied to the treatment of burn patients in a specialized center. Arch Surg 2007;142(1):50-7; discussion 57. 172. Rutten-van Molken MP, Oostenbrink JB, Miravitlles M, Monz BU. Modelling the 5-year cost effectiveness of tiotropium, salmeterol and ipratropium for the treatment of chronic obstructive pulmonary disease in Spain. Eur J Health Econ 2007;8(2):123-35. 173. Rodriguez MJ, Diaz S, Vera-Llonch M, Dukes E, Rejas J. Cost-effectiveness analysis of pregabalin versus gabapentin in the management of neuropathic pain due to diabetic polyneuropathy or post-herpetic neuralgia. Curr Med Res Opin 2007;23(10):2585-96. 174. Pinol C, Roze S, Valentine W, Evers T. [Cost-effectiveness of the addition of acarbose to the treatment of patients with type-2 diabetes in Spain]. Gac Sanit 2007;21(2):97-104; discussion 05. 175. Mercader Cuesta J, Rodríguez Barrios JM, Caro J, Ward J. Impacto económico y sanitario del tratamiento de la bradicardia con marcapasos bicameral vs. monocameral (VVI/R vs. DDD/R). Revista Española de Economía de la Salud 2007;6(5):294-302. 176. Lopez-Bastida J, Soto Pedre E, Cabrera López F. Revisión sistemática y análisis coste-efectividad del cribado de retinopatía diabética con retinógrafo no midriático de 45º mediante imágenes interpretadas por oftalmólogos frente a médicos de familia. Informes de Evaluación de Tecnologías Sanitarias: SESCS 2006/11. Santa Cruz de Tenerife: Servicio de Evaluación del Servicio Canario de la Salud, 2007. 177. Lázaro Y De Mercado P, Figueras J, Domenech E, Closa R, Echániz I, Wood MÁ, et al. Cost-effectiveness of palivizumab in preventing respiratory syncytial virus in premature infants and children with chronic lung disease in Spain. Pharmacoeconomics - Spanish Research Articles 2007;4(2):59-70. 178. Evers SM, Ament AJ, Colombo GL, Konradsen HB, Reinert RR, Sauerland D, et al. Cost-effectiveness of pneumococcal vaccination for prevention of invasive pneumococcal disease in the elderly: an update for 10 Western European countries. Eur J Clin Microbiol Infect Dis 2007;26(8):531-40. 179. Donate J, Mousavi K, Pazos B, Altimiras J, Echarri E, Monte E, et al. Cost-effectiveness analysis of pegaptanib sodium versus photodynamic therapy with verteporfin in the treatment of age-related macular degeneration in Spain. Pharmacoeconomics - Spanish Research Articles 2007;4(4):127-36. 180. Dilla T, Sacristan JA, Rentero ML. Evaluación económica de teriparatida (Forsteo(R)) en el tratamiento de la osteoporosis posmenopáusica. Revista Española de Economía de la Salud 2007;6(1):57-64. 181. Diaz S, Argumosa A, Horga de la Parte JF, Vera-Llonch M, Dukes E, Rejas-Gutierrez J. [Analysis of the cost-effectiveness of treatment for refractory partial epilepsy: a simulation model for pregabalin and levetiracetam]. Rev Neurol 2007;45(8):460-7. 182. Canonica GW, Poulsen PB, Vestenbaek U. Cost-effectiveness of GRAZAX for prevention of grass pollen induced rhinoconjunctivitis in Southern Europe. Respir Med 2007;101(9):1885-94. 183. Aballea S, De Juanes JR, Barbieri M, Martin M, Chancellor J, Oyaguez I, et al. The cost effectiveness of influenza vaccination for adults aged 50 to 64 years: a model-based analysis for Spain. Vaccine 2007;25(39-40):6900-10. 184. Shearer AT, Bagust A, Ampudia-Blasco FJ, Martinez-Lage Alvarez B, Perez Escolano I, Paris G. Lifetime health consequences and cost-effectiveness of rosiglitazone in combination with metformin for the treatment of type 2 diabetes in Spain. Pharmacoeconomics 2006;24 Suppl 1:49-59. 185. Serrano-Blanco A, Pinto-Meza A, Suarez D, Penarrubia MT, Haro JM. Cost-utility of selective serotonin reuptake inhibitors for depression in primary care in Catalonia. Acta Psychiatr Scand Suppl 2006(432):39-47. 186. Serrano Aguilar P, Lopez-Bastida J, Ramallo Fariña Y, Cabrera Hernández J, Perestelo-Pérez L, Garcés Martín G, et al. Análisis coste-efectividad y resultados en salud en cirugía ortopédica de cadera y rodilla. In: Servicio Canario de la Salud CdSdGdC, editor. Informes de Evaluación de Tecnologías Sanitarias: SESCS 2006/16. Santa Cruz de Tenerife: Servicio de Evaluación del Servicio Canario de la Salud, 2006. 187. Rubio Terrés C, Alberola V, Casal J, Felip E, González Larriba J, Álvarez Sanz C. Análisis farmacoeconómico del tratamiento con erlotinib, docetaxel, pemetrexed o tratamiento de soporte de pacientes con cáncer de pulmón no microcítico avanzado, previamente tratado con quimioterapia. Pharmacoeconomics - Spanish Research Articles 2006;3(3):137-49. 188. Mar J, Gutierrez-Moreno S, Chilcott J. [Probabilistic cost-effectiveness analysis of the treatment of sleep apnea]. Gac Sanit 2006;20(1):47-53. 189. Lazaro y de Mercado P, Figueras Aloy J, Domenech Martinez E, Echaniz Urcelay I, Closa Monasterolo R, Wood Wood MA, et al. [The efficiency (cost-effectiveness) of palivizumab as prophylaxis against respiratory syncytial virus infection in premature infants with a gestational age of 32-35 weeks in Spain]. An Pediatr (Barc) 2006;65(4):316-24. 190. Lamotte M, Pinol C, Brotons C, Annemans L, Guardiola E, Evers T, et al. [Health economic evaluation of low-dose acetylsalicylic acid in the primary prevention of cardiovascular disease]. Rev Esp Cardiol 2006;59(8):807-15. 191. Gomez-Outes A, Rocha E, Martinez-Gonzalez J, Kakkar VV. Cost effectiveness of bemiparin sodium versus unfractionated heparin and oral anticoagulants in the acute and long-term treatment of deep vein thrombosis. Pharmacoeconomics 2006;24(1):81-92. 192. Gil JM, Rubio-Terres C, Del Castillo A, Gonzalez P, Canorea F. Pharmacoeconomic analysis of adjuvant therapy with exemestane, anastrozole, letrozole or tamoxifen in postmenopausal women with operable and estrogen receptor-positive breast cancer. Clin Transl Oncol 2006;8(5):339-48. 193. Fernandez de Bobadilla J, Lopez de Sa E, Alonso Troncoso I, Moreno Gomez R, Rubio-Terres C, Soto Alvarez J. [Cost-effectiveness analysis of the use of atorvastatin in patients with type 2 diabetes mellitus: a pharmacoeconomic model of the CARDS study]. An Med Interna 2006;23(5):213-9. 194. Conget Donlo I, Serrano Contreras D, Rodriguez Barrios JM, Levy Mizrahi I, Castell Abat C, Roze S. [Cost-utility analysis of insulin pumps compared to multiple daily doses of insulin in patients with type 1 diabetes mellitus in Spain]. Rev Esp Salud Publica 2006;80(6):679-95. 195. Borgstrom F, Carlsson A, Sintonen H, Boonen S, Haentjens P, Burge R, et al. The cost-effectiveness of risedronate in the treatment of osteoporosis: an international perspective. Osteoporos Int 2006;17(7):996-1007. 196. Rubio-Terres C, Dominguez-Gil Hurle A. [Cost-utility analysis of relapsing-remitting multiple sclerosis treatment with azathioprine or interferon beta in Spain]. Rev Neurol 2005;40(12):705-10. 197. Mar J, Begiristain JM, Arrazola A. Cost-effectiveness analysis of thrombolytic treatment for stroke. Cerebrovasc Dis 2005;20(3):193-200. 198. Grau S, Alvarez-Lerma F, del Castillo A, Neipp R, Rubio-Terres C. Cost-effectiveness analysis of the treatment of ventilator-associated pneumonia with linezolid or vancomycin in Spain. J Chemother 2005;17(2):203-11. 199. Cannata J, Díez A, López J. Estudio de coste-efectividad de protelos en la prevención de fracturas osteoporóticas. Revista Española de Economía de la Salud 2005;4(3):146-52. 200. Medina-Redondo F, Herrera-Carranza J, Sanabria C, Navarro G, Garcia-Moreno JM, Gamero-Garcia MA, et al. [The efficiency and cost-utility ratio of interferon beta in the treatment of multiple sclerosis in Andalusia]. Rev Neurol 2004;39(1):1-6. 201. Latour-Perez J, Navarro-Ruiz A, Ridao-Lopez M, Cervera-Montes M. Using clopidogrel in non-ST-segment elevation acute coronary syndrome patients: a cost-utility analysis in Spain. Value Health 2004;7(1):52-60. 202. San Miguel R, Mar J, Cabases JM, Guillen-Grima F, Buti M. Cost-effectiveness analysis of therapeutic strategies for patients with chronic hepatitis C previously not responding to interferon. Aliment Pharmacol Ther 2003;17(6):765-73. 203. Rubio-Terres C, Aristegui Ruiz I, Medina Redondo F, Izquierdo Ayuso G. [Cost-utility analisys of multiple sclerosis treatment with glatiramer acetate or interferon beta in Spain]. Farm Hosp 2003;27(3):159-65. 204. Rubio Terrés C, Rodríguez J, Bolinder B, Pablos Pd. Análisis coste-utilidad del tratamiento de la diabetes mellitus con insulina glargina o insulina NPH en España. Revista Española de Economía de la Salud 2003;2(6):313-24. 205. Mar J, Rueda JR, Duran-Cantolla J, Schechter C, Chilcott J. The cost-effectiveness of nCPAP treatment in patients with moderate-to-severe obstructive sleep apnoea. Eur Respir J 2003;21(3):515-22. 206. García de Villaescusa Collazo R, Barallobre J, Staginnus U. Coste efectividad de las transfusiones de componentes plaquetarios preparados con tratamiento de inactivación de patógenos en España. Revista Española de Economía de la Salud 2003;2(3):166-75. 207. Gambús G, Espinosa C. Análisis coste-efectividad de fluvastatina en pacientes coronarios tras una intervención coronaria percutánea. Revista Española de Economía de la Salud 2003;2(6):327-29. 208. Hart W, Rubio-Terrés C, Burrell A, Aristegui I, Escobar-Jiménez F. Análisis farmacoeconómico del tratamiento de la osteoporosis postmenopáusica con risedronato o alendronato. Revista Española de Enfermedades Metabólicas Oseas 2002;11(3):97-104. 209. Escudero Pereira J, Bouzas Caamaño E, Domínguez Hernández V, Farjas Abadía M, Lema Devesa M. Infección neumocócica en el Complejo Universitario Juan Canalejo: análisis coste/beneficio de una política de inmunización al alta hospitalaria. Vacunas 2002;3(3):94-8. 210. Cordobés J, Corominas C, Díaz M, Gómez F, Juliá J, Lozano P, et al. Coste utilidad y calidad de vida del tratamiento endovascular en el sector ilíaco. Archivos de Cirugía Vascular 2002;11(1):3-8. 211. Alonso Cuesta P, López Martínez J, del Nogal Sáez F, Jiménez Martín M, Rodriguez Roldan J, Suárez Saiz J. Análisis de coste-efectividad de la ventilación mecánica y del tratamiento intensivo de pacientes en situación crítica. Medicina Intensiva 2002;26(8):391-98. 212. Mar J, Rodriguez-Artalejo F. Which is more important for the efficiency of hypertension treatment: hypertension stage, type of drug or therapeutic compliance? J Hypertens 2001;19(1):149-55. 213. Tarraga Lopez PJ, Marin Nieto E, Celada Rodriguez A, Garcia Molinero MJ, Garcia Olmo D, Solera Albero J. Economic evaluation of colorectal cancer screening with fecal occult blood detection. Rev Esp Enferm Dig 2000;92(5):334-48. 214. Buti M, Casado MA, Fosbrook L, Wong JB, Esteban R. Cost-effectiveness of combination therapy for naive patients with chronic hepatitis C. J Hepatol 2000;33(4):651-8. 215. Ament A, Baltussen R, Duru G, Rigaud-Bully C, de Graeve D, Ortqvist A, et al. Cost-effectiveness of pneumococcal vaccination of older people: a study in 5 western European countries. Clin Infect Dis 2000;31(2):444-50. 216. Pereira A. Cost-effectiveness of transfusing virus-inactivated plasma instead of standard plasma. Transfusion 1999;39(5):479-87. 217. Anon JM, Garcia de Lorenzo A, Zarazaga A, Gomez-Tello V, Garrido G. Mechanical ventilation of patients on long-term oxygen therapy with acute exacerbations of chronic obstructive pulmonary disease: prognosis and cost-utility analysis. Intensive Care Med 1999;25(5):452-7. 218. Pardo Mateu L, Alfonso Sánchez J. Aplicación del análisis de coste-utilidad al tratamiento de los pacientes quemados, en una unidad especializada. Cirugía Plástica Ibero-Latinoamericana 1998;24(4):425-35. 219. Buti M, Casado MA, Fosbrook L, Esteban R. [Cost effectiveness of the treatment of chronic hepatitis C with interferon-alpha]. Gastroenterol Hepatol 1998;21(4):161-8. 220. Plans P, Casademont L, Tarin A, Navas E. [The study of cost effectiveness of breast cancer detection in Catalonia]. Rev Esp Salud Publica 1996;70(1):15-23. 221. Plans Rubio P, Garrido Morales P, Salleras Sanmarti L. [The cost-effectiveness of pneumococcal vaccination in Catalonia]. Rev Esp Salud Publica 1995;69(5):409-17. 222. Jonsson B, Horisberger B, Bruguera M, Matter L. Cost-benefit analysis of hepatitis-B vaccination. A computerized decision model for Spain. Int J Technol Assess Health Care 1991;7(3):379-402. 223. Rovira J, Lobo F, Badia X, Ventura J, de la Fuente M, Brosa M. Aproximación a un análisis coste-utilidad de la terapéutica transfusión/eritropoyetina en los pacientes afectos de anemia nefrológica en hemodiálisis: resultados del estudio en curso. SEDYT 1989;11:15-20. |
| --- |

**Table S3**: PRISMA Checklist

| **Section/topic** | **#** | **Checklist item** | **Reported on page #** |
| --- | --- | --- | --- |
| **TITLE** | | |  |
| Title | 1 | Identify the report as a systematic review, meta-analysis, or both. | Page 1 |
| **ABSTRACT** | | |  |
| Structured summary | 2 | Provide a structured summary including, as applicable: background; objectives; data sources; study eligibility criteria, participants, and interventions; study appraisal and synthesis methods; results; limitations; conclusions and implications of key findings; systematic review registration number. | Page 2 |
| **INTRODUCTION** | | |  |
| Rationale | 3 | Describe the rationale for the review in the context of what is already known. | Page 3 |
| Objectives | 4 | Provide an explicit statement of questions being addressed with reference to participants, interventions, comparisons, outcomes, and study design (PICOS). | Page 3 (not PICO, this is a methodological review) |
| **METHODS** | | |  |
| Protocol and registration | 5 | Indicate if a review protocol exists, if and where it can be accessed (e.g., Web address), and, if available, provide registration information including registration number. | Page 3 |
| Eligibility criteria | 6 | Specify study characteristics (e.g., PICOS, length of follow-up) and report characteristics (e.g., years considered, language, publication status) used as criteria for eligibility, giving rationale. | Page 4 |
| Information sources | 7 | Describe all information sources (e.g., databases with dates of coverage, contact with study authors to identify additional studies) in the search and date last searched. | Page 3 |
| Search | 8 | Present full electronic search strategy for at least one database, including any limits used, such that it could be repeated. | Table S1 (appendix) |
| Study selection | 9 | State the process for selecting studies (i.e., screening, eligibility, included in systematic review, and, if applicable, included in the meta-analysis). | Page 4-5 |
| Data collection process | 10 | Describe method of data extraction from reports (e.g., piloted forms, independently, in duplicate) and any processes for obtaining and confirming data from investigators. | Page 4-5 |
| Data items | 11 | List and define all variables for which data were sought (e.g., PICOS, funding sources) and any assumptions and simplifications made. | Page 4-5 (Tables 1-4) |
| Risk of bias in individual studies | 12 | Describe methods used for assessing risk of bias of individual studies (including specification of whether this was done at the study or outcome level), and how this information is to be used in any data synthesis. | NA (no risk of bias tool for cost-effectiveness analyses) |
| Summary measures | 13 | State the principal summary measures (e.g., risk ratio, difference in means). | NA  (not meta-analysis) |
| Synthesis of results | 14 | Describe the methods of handling data and combining results of studies, if done, including measures of consistency (e.g., I^2^) for each meta-analysis. | NA  (not meta-analysis) |

| Risk of bias across studies | 15 | Specify any assessment of risk of bias that may affect the cumulative evidence (e.g., publication bias, selective reporting within studies). | NA  (not meta-analysis) |
| --- | --- | --- | --- |
| Additional analyses | 16 | Describe methods of additional analyses (e.g., sensitivity or subgroup analyses, meta-regression), if done, indicating which were pre-specified. | NA  (not meta-analysis) |
| **RESULTS** | | |  |
| Study selection | 17 | Give numbers of studies screened, assessed for eligibility, and included in the review, with reasons for exclusions at each stage, ideally with a flow diagram. | Page 5-6  (flowchart Fig S1 in appendix) |
| Study characteristics | 18 | For each study, present characteristics for which data were extracted (e.g., study size, PICOS, follow-up period) and provide the citations. | Page 5-6  (Tables 1-4) |
| Risk of bias within studies | 19 | Present data on risk of bias of each study and, if available, any outcome level assessment (see item 12). | NA (no risk of bias tool for cost-effectiveness analyses) |
| Results of individual studies | 20 | For all outcomes considered (benefits or harms), present, for each study: (a) simple summary data for each intervention group (b) effect estimates and confidence intervals, ideally with a forest plot. | NA  (not meta-analysis) |
| Synthesis of results | 21 | Present results of each meta-analysis done, including confidence intervals and measures of consistency. | NA  (not meta-analysis) |
| Risk of bias across studies | 22 | Present results of any assessment of risk of bias across studies (see Item 15). | NA  (not meta-analysis) |
| Additional analysis | 23 | Give results of additional analyses, if done (e.g., sensitivity or subgroup analyses, meta-regression [see Item 16]). | NA  (not meta-analysis) |
| **DISCUSSION** | | |  |
| Summary of evidence | 24 | Summarize the main findings including the strength of evidence for each main outcome; consider their relevance to key groups (e.g., healthcare providers, users, and policy makers). | Page 7-9 |
| Limitations | 25 | Discuss limitations at study and outcome level (e.g., risk of bias), and at review-level (e.g., incomplete retrieval of identified research, reporting bias). | Page 9-10 |
| Conclusions | 26 | Provide a general interpretation of the results in the context of other evidence, and implications for future research. | Page 10 |
| **FUNDING** | | |  |
| Funding | 27 | Describe sources of funding for the systematic review and other support (e.g., supply of data); role of funders for the systematic review. | Page 1 |

*From:*  Moher D, Liberati A, Tetzlaff J, Altman DG, The PRISMA Group (2009). Preferred Reporting Items for Systematic Reviews and Meta-Analyses: The PRISMA Statement. PLoS Med 6(6): e1000097. doi:10.1371/journal.pmed1000097

For more information, visit: **www.prisma-statement.org**.

**Figure S1. Flow diagram for study selection.**

**Figure S2. Growth in published cost-effectiveness analyses. Spain, 1989-2014.**
